# Supplementary material for: Antisolvent controls the shape and size of anisotropic lead halide perovskite nanocrystals
Source: Nat Commun. 2024 Oct 17;15:8952. doi: 10.1038/s41467-024-53221-5 (PMC11486954; doi:10.1038/s41467-024-53221-5)
Supplement: Supplementary file 1 — Supplementary Information [file 41467_2024_53221_MOESM1_ESM.pdf]

Supplementary Information for:
Antisolvent controls the shape and size of anisotropic lead halide perovskite nanocrystals

September 2024

Author list

Kilian Frank[1,#], Nina A. Henke[2,#], Carola Lampe[2], Tizian Lorenzen[3], Benjamin März[3], Xiao Sun[4], Sylvio Haas[4], Olof Gutowski[4], Ann-Christin Dippel[4], Veronika Mayer[2] Knut Müller-Caspary[3], Alexander S. Urban[2,*], Bert Nickel[1,*]

Affiliations

[1] K. Frank, B. Nickel
Soft Condensed Matter Group and Center for NanoScience
Faculty of Physics
Ludwig-Maximilians-Universität München
Geschwister-Scholl-Platz 1, 80539 Munich, Germany

[2] N. A. Henke, C. Lampe, V. Mayer, A. S. Urban
Nanospectroscopy Group and Center for NanoScience
Faculty of Physics
Ludwig-Maximilians-Universität München
Königinstraße 10, 80539 Munich, Germany

[3] T. Lorenzen, B. März, K. Müller-Caspary
Department of Chemistry and Center for NanoScience
Ludwig-Maximilians-Universität München
Butenandtstraße 11, 81377 Munich, Germany

[4] X. Sun, S. Haas, O. Gutowski, A.-C. Dippel
Deutsches Elektronen-Synchrotron DESY
Notkestraße 85, 22607 Hamburg, Germany

[#] These authors contributed equally: Kilian Frank, Nina A. Henke.
[*] email: urban@lmu.de, nickel@lmu.de

Contents

1 Nanocrystal size distributions and ex situ characterisation **4**
Supplementary Fig. 1 - Size distribution of $CsPbBr_3$ 3ML nanorods and 2ML nanoplatelets. 4
Supplementary Fig. 2 - ADF-STEM imaging of perovskite nanorods and nanoplatelets. 4
Supplementary Fig. 3 - Ex situ absorbance and photoluminescence spectra of perovskite nanocrystals. . 5
Supplementary Fig. 4 - Analysis of potential byproducts by PXRD. 5
Supplementary Fig. 5 - PLE spectra of purified perovskite nanocrystals. 6
Supplementary Fig. 6 - SAXS analysis of purified and resuspended 2ML nanoplatelets and 3ML nanorods measured with a laboratory setup. 6
Supplementary Table 1 - SAXS fit parameters for nanoplatelets and nanorods measured with a laboratory setup. 6

2 In situ WAXS and total scattering **7**
Supplementary Fig. 7 - In situ reaction cell. 7
Supplementary Table 2 - Parameters of the PDF transformation. 7
Supplementary Fig. 8 - WAXS intensities of a synthesis of 3ML nanorods. 8
Supplementary Fig. 9 - WAXS analysis of the (040) and (202) reflections of a 3ML nanorod synthesis. . 8
Supplementary Fig. 10 - Analysis of potential intermediates or byproducts by WAXS. 9
Supplementary Fig. 11 - PL analysis after Cs-oleate injection and after antisolvent injection. 9

3 In situ SAXS **9**
Supplementary Table 3 - Scattering length densities of components used in SAXS analysis. 10
Supplementary Fig. 12 - Comparison of the PL of intermediate nanoclusters, 2ML nanoplatelets, and 3ML nanorods. 10
Supplementary Fig. 13 - SAXS analysis of the $PbBr_2$- and the Cs-oleate precursor. 10
Supplementary Table 4 - SAXS fit parameters of the precursors. 11
Supplementary Table 5 - Number of precursor micelles involved in intermediate nanocluster nucleation. . 11
Supplementary Fig. 14 - Absorbance spectra of $PbBr_2$precursor. 11
Supplementary Fig. 15 - WAXS and PDF analysis of the $PbBr_2$ precursor. 12
Supplementary Fig. 16 - Comparison of different shape models of the intermediate nanoclusters. 14
Supplementary Table 6 - SAXS fit parameters for different shape models for 3ML nanorod synthesis. . . . 15
Supplementary Table 7 - SAXS fit parameters for different shape models for 2ML nanoplatelet synthesis. 16
Supplementary Fig. 17 - SAXS fit results using a model of polydisperse spheres, or cuboids with two identical sides. 17
Supplementary Fig. 18 - SAXS analysis of intermediate nanocluster formation for a 3ML nanorod synthesis. 17
Supplementary Fig. 19 - Evolution of number densities of precursor micelles and intermediate nanoclusters. 18
Supplementary Fig. 20 - Optical characterisation and TEM imaging of a precursor mixture containing perovskite intermediate nanoclusters. 18
Supplementary Table 8 - Critical synthesis parameters used in the in situ experiments. 19
Supplementary Fig. 21 - SAXS data after antisolvent injection. 20
Supplementary Fig. 22 - SAXS analysis of the mesophase reflections. 21

4 Variation of precursor concentration and antisolvent volume **22**
Supplementary Fig. 23 - Comparison of synthesis products at different precursor concentrations and antisolvent volumes. 22
Supplementary Fig. 24 - SAXS data of syntheses without $PbBr_2$ or Cs-oleate. 23
Supplementary Fig. 25 - In situ PL of 2ML nanoplatelet syntheses with varying volumes of acetone antisolvent. 24
Supplementary Table 9 - Exponential association fit parameters. 24
Supplementary Fig. 26 - In situ PL and ex situ optical and morphological characterisation of syntheses without antisolvent. 25
Supplementary Fig. 27 - In situ PL and growth kinetics of 2ML nanoplatelets synthesised without antisolvent at different precursor concentrations. 26
Supplementary Table 10 - Biphase exponential association fit parameters. 27
Supplementary Fig. 28 - Variation of antisolvent properties controls the dimensionality and thickness of anisotropic $CsPbBr_3$ nanocrystals. 27

5 Characterisation of 3ML nanoplatelets **28**
Supplementary Fig. 29 - Optical properties, morphology and assembly of 3ML $CsPbBr_3$ nanoplatelets. . 28

6 Antisolvent polarity and classification **28**
Supplementary Table 11 - Polarity of antisolvents. 29
Supplementary Table 12 - Parameters for the ex situ synthesis of monodisperse $CsPbBr_3$ nanoplatelets and nanorods. 30

7 Tuning of color, size, and aspect ratio **31**
Supplementary Fig. 30 - Optical properties and morphology of $CsPbBr_3$ nanocrystals synthesised with various ketones as moderate antisolvents. 31
Supplementary Fig. 31 - Optical properties of $CsPbBr_3$ nanocrystals obtained by antisolvent variation in room temperature synthesis. 32
Supplementary Fig. 32 - Tunable aspect ratio of 3ML nanorods. 33
Supplementary Fig. 33 - Tunable lateral sizes of 2ML nanoplatelets. 34
Supplementary Fig. 34 - Photoluminescence quantum yield of $CsPbBr_3$ nanoplatelets and nanorods. . . . 35
Supplementary Fig. 35 - Colloidal stability of perovskite nanoplatelets and nanorods. 36
Supplementary Fig. 36 - Influence of Cs/Pb, Cs/Br and Pb/Br ratio on nanocrystal product and emission wavelength. 37

8 Chemicals **38**
Supplementary Table 13 - Chemicals. 38

1 Nanocrystal size distributions and ex situ characterisation

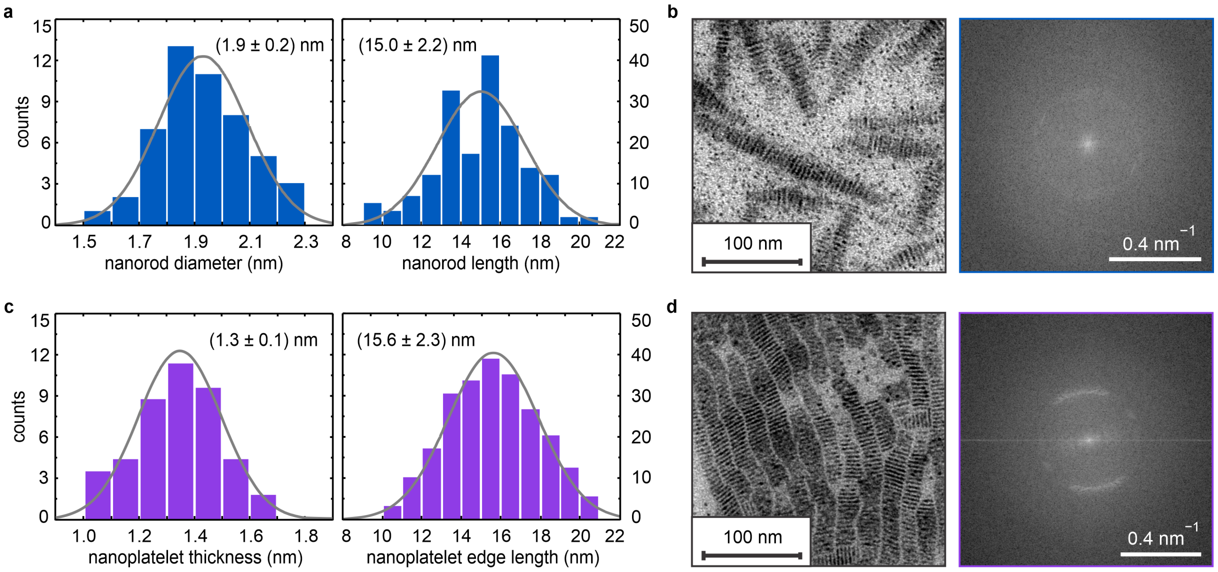

Supplementary Fig. 1 | Size distribution of CsPbBr$_3$ 3ML nanorods and 2ML nanoplatelets. **a**, 3 monolayer (ML) nanorods have an average diameter of (1.9 ± 0.2) nm and a length of (15.0 ± 2.2) nm, also shown in Fig. 1 c. **b**, Transmission electron microscopy (TEM) image of assembled nanorods and the respective Fast Fourier transform (FFT) image, confirming a regular spacing (4.83 nm) of 3ML nanorods. Nanorods lay flat on the TEM substrate. **c**, 2ML nanoplatelets have an average thickness of (1.3 ± 0.1) nm and square lateral dimensions of (15.6 ± 2.3) nm, as shown in Fig. 1 c. **d**, TEM image of stacks of edge-up oriented 2ML nanoplatelets and the respective FFT image, confirming a regular stacking distance (4.16 nm) of 2ML nanoplatelets.

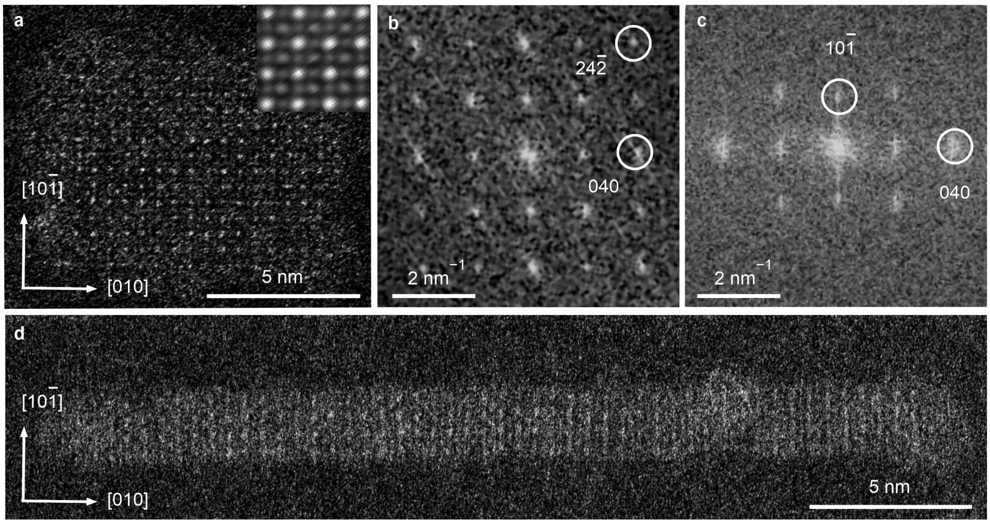

Supplementary Fig. 2 | ADF-STEM imaging of perovskite nanorods and nanoplatelets. **a**, Annular dark field scanning transmission electron microscopy (ADF-STEM) image of a CsPbBr$_3$ nanoplatelet with indexed directions. Here a [101] zone axis was assumed. The inset shows a Wiener filtered unit cell average. **b**, Power spectrum of **a** with indexed reciprocal space directions and two indexed reflections ((0 4 0), (2 4 $\bar{2}$)). **c**, Power spectrum with two indexed reflections ((1 0 $\bar{1}$), (0 4 0)) of a CsPbBr$_3$ nanorod shown in **d**. Images along the [0 1 0] and [1 0 1] zone axis of the orthorhombic CsPbBr$_3$ and the [0 0 1] zone axis of the cubic CsPbBr$_3$ show a high similarity making it difficult to distinguish the three projections in ADF-STEM imaging. Crystal directions were assigned for the orthorhombic perovskite crystal structure in agreement with the results from powder X-ray diffraction measurements in Supplementary Fig. 4.

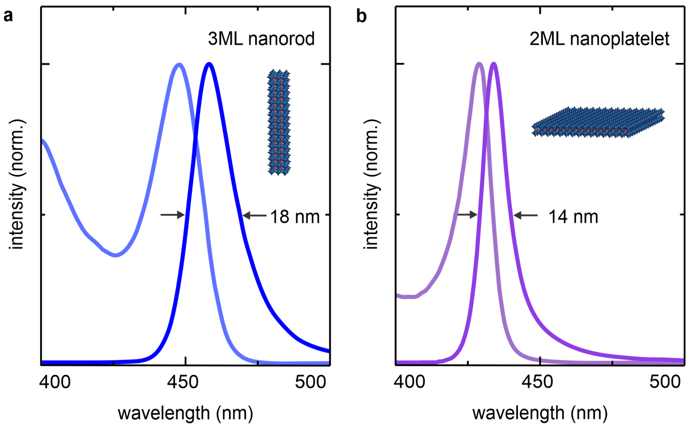

Supplementary Fig. 3 | Ex situ absorbance and photoluminescence of perovskite nanocrystals. **a**, 3 monolayer (ML) $CsPbBr_3$ nanorods with excitonic absorption at 448 nm and photoluminescence (PL) at 460 nm, full width at half maximum (FWHM) = 18 nm. **b**, 2ML $CsPbBr_3$ nanoplatelets with excitonic absorption at 428 nm and PL at 434 nm (FWHM = 14 nm).

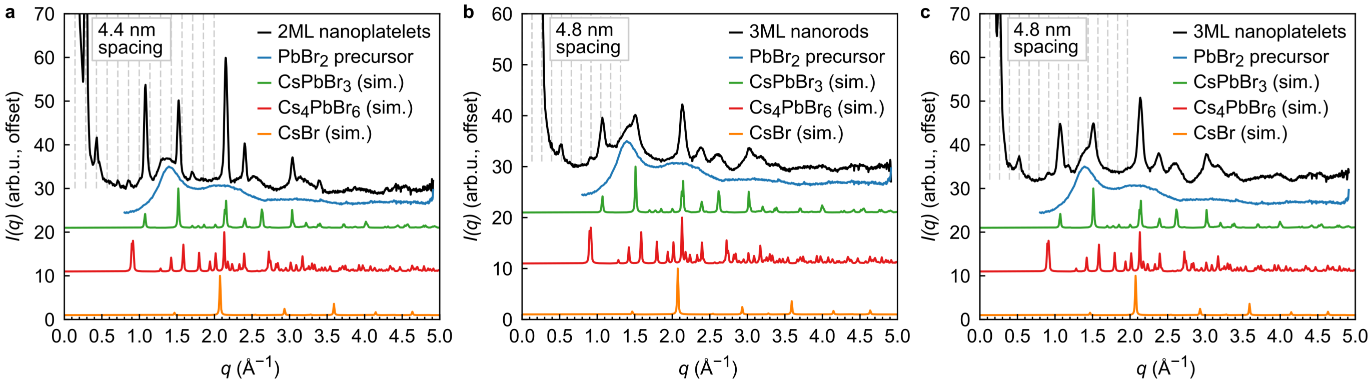

Supplementary Fig. 4 | Analysis of potential byproducts by PXRD. **a**, Powder X-ray diffraction (PXRD) data of purified and dropcasted 2 monolayer (ML) nanoplatelets (**a**), 3ML nanorods (**b**), and 3ML nanoplatelets (**c**) (black). The powder diffractogram of dried $PbBr_2$ precursor solution (blue) and simulated intensities of $CsPbBr_3$, Cs_4PbBr_6, and CsBr are shown in all subfigures for comparison. Vertical dashed lines indicate the spacing between nanocrystals resulting from self-assembly upon drying of the dropcasted sample. 2ML nanoplatelets show a regular series of reflections at low q which correspond to a spacing of 4.4 nm. 3ML nanorods and 3ML nanoplatelets show a spacing of 4.8 nm. Both are in agreement with the respective nanocrystal thickness plus a double layer of oleylamine/oleic acid ligands (3 nm). At larger q, the signal of nanocrystalline $CsPbBr_3$ dominates. A broad reflection at 1.4 $Å^{-1}$ likely comes from excess ligands, since a similar signal is also observed in the dried $PbBr_2$ precursor solution, and reported in literature for amorphous oleylamine[1]. No additional Cs_4PbBr_6 or CsBr have to be taken into account to describe the patterns, showing the absence of these potential byproducts. The diffraction patterns were simulated using Mercury[2] based on the Crystallography Open Database (COD)[3] entries 4510745 for orthorhombic (Pnma) $CsPbBr_3$, 1538416 for trigonal ($R\bar{3}c$:H) Cs_4PbBr_6, and 9008788 for cubic ($Pm\bar{3}m$) CsBr.

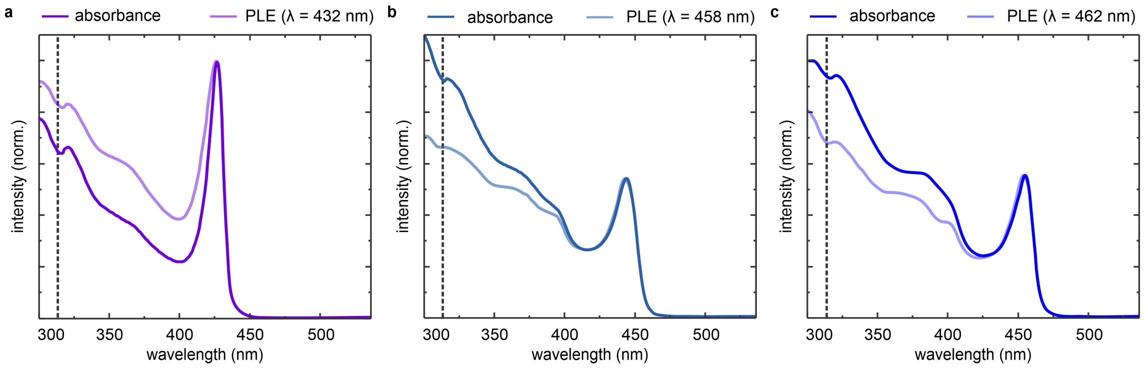

Supplementary Fig. 5 | Photoluminescence excitation spectra of purified perovskite nanocrystals. The absorbance of as-synthesised **a**, 2 monolayer (ML) nanoplatelets **b**, 3ML nanorods and **c**, 3ML nanoplatelets shows good agreement with respective photoluminescence excitation. For comparison, the characteristic absorption feature of lead-deficient, non-emissive Cs_4PbBr_6 at 315 nm is marked with a grey dotted line.

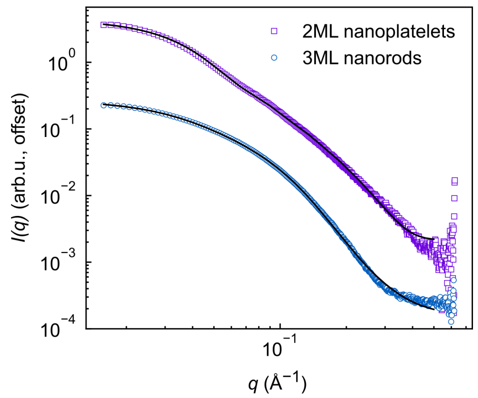

Supplementary Fig. 6 | SAXS analysis of purified and resuspended 2ML nanoplatelets and 3ML nanorods measured with a laboratory setup. The small-angle X-ray scattering (SAXS) intensity of purified anisotropic nanocrystals in n-hexane is described very well by an ellipsoid model (black lines), detailed in Supplementary Note 3. The oblate shape of 2 monolayer (ML) nanoplatelets, (1.7 × 14.6 × 14.6) nm^3 is clearly confirmed. 3ML nanorods are instead found to be prolate (2.1 × 2.1 × 17.1) nm^3. All fit parameters are given in Supplementary Table 1.

Supplementary Table 1 | SAXS fit parameters for nanoplatelets and nanorods measured with a laboratory setup. Uncertainties are provided by the Differential Evolution Adaptive Metropolis (DREAM) algorithm of SasView and correspond to 68 % confidence intervals. The scattering length densities of particle and solvent were set to $32 \cdot 10^{-6}$ Å^{-2} and $6.5 \cdot 10^{-6}$ Å^{-2}, respectively (Supplementary Table 3). Relative polydispersity (rel. PD) is defined as the ratio of standard deviation and mean $\frac{\sigma_l}{l}$ or $\frac{\sigma_d}{d}$ of a Gaussian distribution of sizes.

	**2ML nanoplatelets**	**3ML nanorods**	**3ML nanoplatelets**
scale (arb.u.)	$(7.11 \pm 0.02) \cdot 10^{-4}$	$(1.774 \pm 0.002) \cdot 10^{-3}$	$(1.50 \pm 0.02) \cdot 10^{-4}$
background (arb.u.)	$(3.67 \pm 0.07) \cdot 10^{-3}$	$(7.82 \pm 0.08) \cdot 10^{-3}$	$(7.9 \pm 0.3) \cdot 10^{-4}$
length l (nm) (rel. PD in brackets)	1.724 ± 0.005	17.07 ± 0.07 (0.18 ± 0.02)	2.15 ± 0.03
diameter d (nm) (rel. PD in brackets)	14.60 ± 0.02	2.05 ± 0.03 (0.69 ± 0.02)	10.38 ± 0.06
χ^2	5.5646	5.264	1.1786

2 In situ WAXS and total scattering

2.1 WAXS analysis

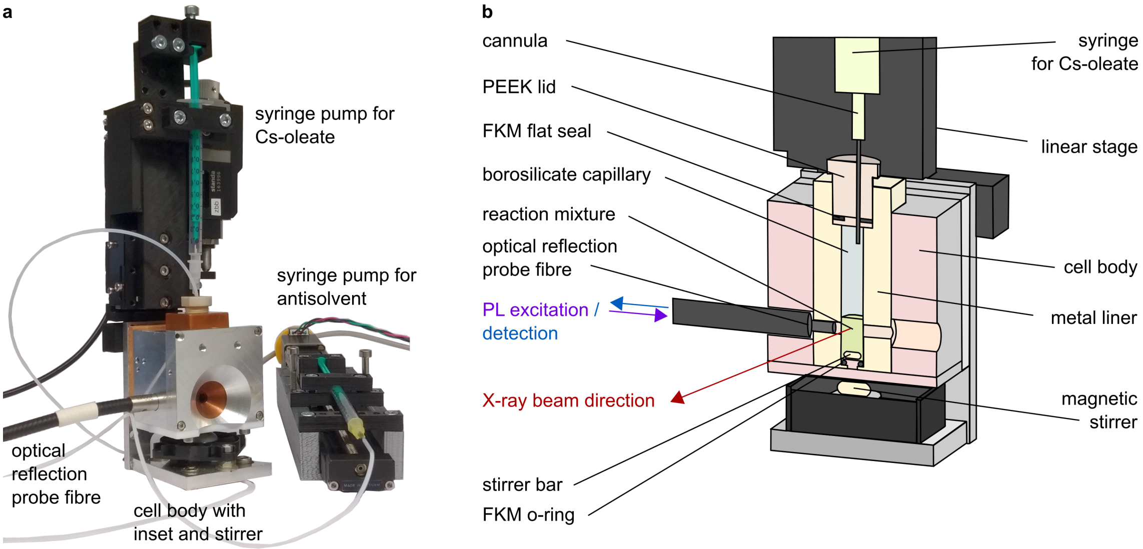

Supplementary Fig. 7 | In situ reaction cell. a, Photograph of the reaction cell with connected syringe pumps and optical reflection probe fibre. **b**, Cross-section view of the cell body containing a metal liner with a borosilicate capillary of 6.4 mm internal diameter and 0.05 mm wall thickness. The reaction mixture is stirred by a magnetic stirrer bar. Cs-oleate is injected through an opening in the lid. The X-ray beam traverses the cell perpendicular to the optical reflection probe fibre. Photoluminescence is recorded in reflection geometry.

2.2 Pair distribution function analysis

Selected in situ WAXS data were Fourier-transformed to the reduced pair distribution function (PDF) $G(r)$ using PDFgetX3[4]. $G(r)$ is related to the background-subtracted intensity $I(q)$ via the structure function $S(q)$ and the reduced structure function $F(q)$[5]:

$$S(q) = \frac{I(q)}{N\langle f(q)\rangle^2} + \frac{\langle f(q)\rangle^2 - \langle f(q)^2\rangle}{\langle f(q)\rangle^2} \tag{S1}$$

$$F(q) = q\,[S(q) - 1] \tag{S2}$$

$$G(r) = \frac{2}{\pi}\int_{q_{\mathrm{min}}}^{q_{\mathrm{max}}} F(q)\sin(qr)dq \tag{S3}$$

N is the number of scattering atoms and $f(q)$ is the atomic form factor. In the procedure a polynomial background, determined by the parameters $q_{\mathrm{max,inst}}$ and r_{poly}, is removed from the data. All parameters are given in Supplementary Table 2.

Supplementary Table 2 | Parameters of the PDF transformation.

$q_{\mathrm{min}}(\mathrm{\AA}^{-1})$	$q_{\mathrm{max}}(\mathrm{\AA}^{-1})$	$q_{\mathrm{max,inst}}(\mathrm{\AA}^{-1})$	$r_{\mathrm{poly}}(\mathrm{\AA})$
1.8	14.3	18.2	1.23

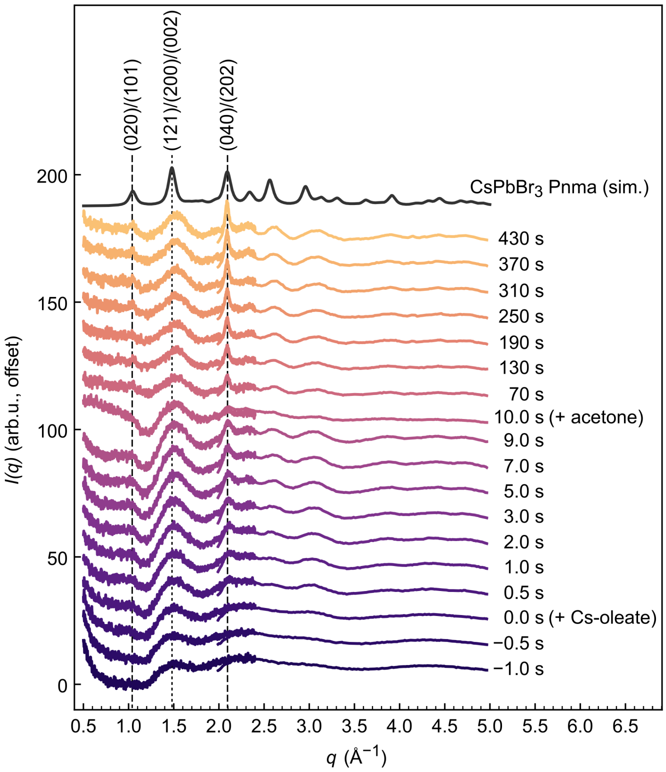

Supplementary Fig. 8 | WAXS intensities of a synthesis of 3ML nanorods. Data are shown for selected reaction times (see labels), with $t = 0\,\mathrm{s}$ corresponding to the Cs-oleate injection. A second Varex Imaging XRD 4343CT detector placed at 4.617 m was used to record the low-q data. Data from the two detectors overlap in the range between 2.0 and 2.4 $\mathrm{\AA}^{-1}$. Starting from the Cs-oleate injection, pronounced Bragg reflections of $CsPbBr_3$ start to appear above the broad signal of the $PbBr_2$ precursor. The (020)/(101) and (040)/(202) reflections of $CsPbBr_3$ are highlighted with vertical dashed lines. The (121)/(200)/(002) reflection (dotted line) is close to the $PbBr_2$ signal and was therefore not analysed. At $t = 10\,\mathrm{s}$, acetone is injected as an antisolvent. As a result, the intensity of $CsPbBr_3$ drops, but later recovers ($t = 70 - 550\,\mathrm{s}$). A simulated wide-angle X-ray scattering (WAXS) intensity of orthorhombic $CsPbBr_3$ (COD 4510745)[6,3] is shown for comparison. The intensity was simulated using Mercury[2].

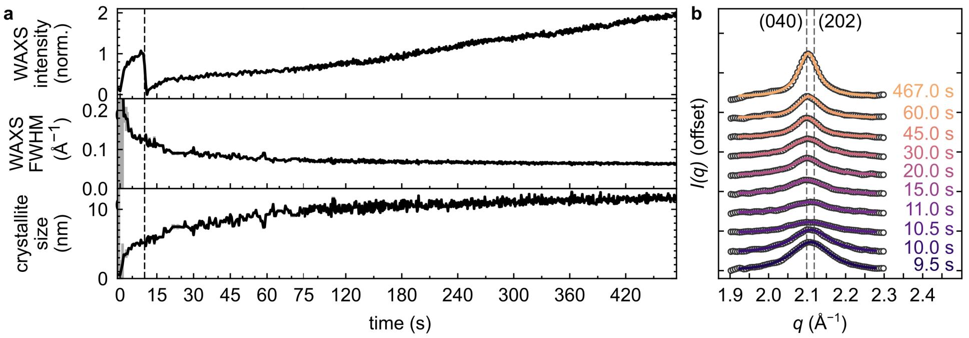

Supplementary Fig. 9 | WAXS analysis of the (040) and (202) reflections of a 3ML nanorod synthesis. **a**, After Cs-oleate injection ($t = 0\,\mathrm{s}$), wide-angle X-ray scattering (WAXS) intensity rises, the reflections become sharper, and the domain size extracted from WAXS indicates the formation of intermediate nanoclusters. After acetone injection, PL and WAXS intensity are reduced ($t = 10\,\mathrm{s}$, vertical dashed line). The strong decrease in WAXS intensity, exceeding the effect of dilution, indicates the dissolution of some nanoclusters. The domain size remains unaffected by antisolvent addition and continues to increase as intermediate nanoclusters start to fuse and recrystallise within the hexagonal mesophase. The domain size then further grows and saturates at a value of (11.7 ± 0.2) nm, corresponding approximately to the long axis of 3 monolayer (ML) nanorods. Error bars denote the parameter uncertainty obtained from a fit with a Pseudo-Voigt peak on a linear background. **b**, Selected WAXS data (dots) and fits to the data (solid lines).

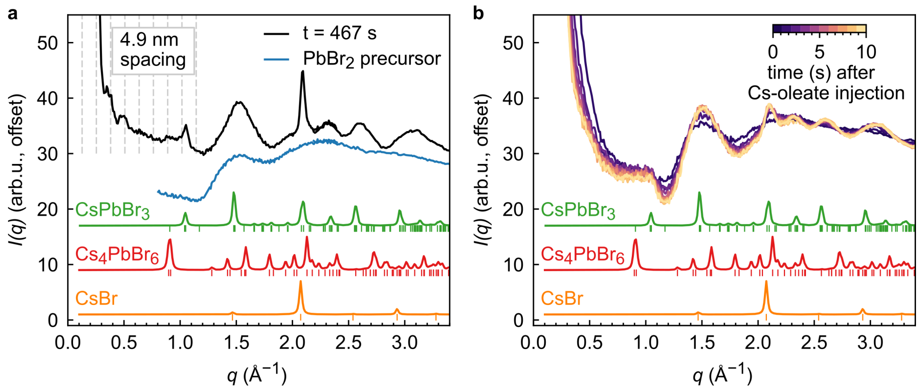

Supplementary Fig. 10 | Analysis of potential intermediates or byproducts by WAXS. **a**, In situ small- and wide-angle X-ray scattering (SAXS/WAXS) intensity of the late stage (467 s) of a 3 monolayer (ML) nanorod synthesis (black) and of the initial $PbBr_2$ precursor (blue). Simulated intensities of $CsPbBr_3$, Cs_4PbBr_6, and CsBr are shown for comparison. Grey dashed lines indicate a spacing of 4.9 nm resulting from the packing of nanocrystals inside the mesophase. The full signal of the nanorod synthesis can be explained by $CsPbBr_3$ nanocrystals and broad peaks from excess ligands, which are also present in the $PbBr_2$ precursor. Additional Cs_4PbBr_6 or CsBr is not detectable at this stage. **b**, In situ SAXS/WAXS data of the early stage of a 3ML nanorod synthesis in between Cs-oleate injection ($t = 0$ s) and acetone injection ($t = 10$ s, color code in legend). Also at this stage no Cs_4PbBr_6 or CsBr is detected. In particular, the reflections of Cs_4PbBr_6 at 0.9 Å^{-1} and 1.8 Å^{-1} are not observed. The reflections of $CsPbBr_3$ at 2.09 Å^{-1} and 2.96 Å^{-1} do not markedly extend to lower q-values, where CsBr reflections would be expected. Therefore, we do not find Cs_4PbBr_6 or CsBr as intermediate phases in the synthesis.

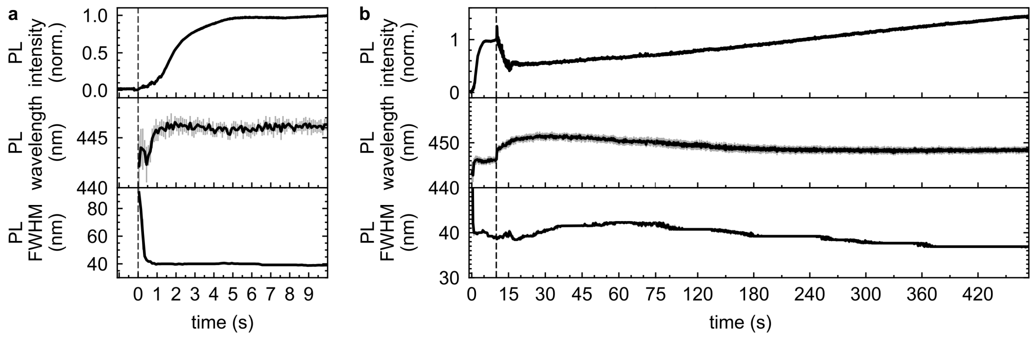

Supplementary Fig. 11 | PL analysis after Cs-oleate injection and after antisolvent injection. **a**, Photoluminescence (PL) emission wavelength and full width at half maximum (FWHM) of the reaction mixture quickly stabilise at 446 nm and 40 nm, respectively, after the Cs-oleate injection (vertical dashed line). **b**, After antisolvent injection (vertical dashed line) the PL intensity grows linearly with time after an initial drop, emission redshifts to 451 nm, followed by a slow blueshift to 448 nm. Meanwhile, the FWHM slightly broadens to 42 nm followed by a narrowing to 37 nm. This indicates that while intermediate nanoclusters fuse to nanorods the size distribution is shortly broadened, before an equilibrium nanorod length is reached. Error bars show the standard deviation of 20 frames of 50 ms each.

3 In situ SAXS

3.1 Intensity model of the precursors

The $PbBr_2$ precursor and Cs-oleate were modelled as core-shell-spheres, i.e., micelles with a $PbBr_2$ or Cs core and an organic ligand shell, interacting via a hard sphere interaction. Assumed scattering length densities (SLDs) of core ($PbBr_2$) and solvent (toluene) are given in Supplementary Table 3. For Cs-oleate, SLDs of 1 and 0 were assumed for core and solvent, as these data were not on an absolute scale, and only the difference of core and solvent SLD enters the calculation. The radius of the core, the thickness and density of the shell, the volume fraction of core-shell-spheres, the scale factor and a constant background were allowed to vary (6 free parameters). The fits to the data are shown in Supplementary Fig. 13, and fit parameters are given in Supplementary Table 4. The micellar structure of Cs-oleate suggested by the model fits has also been reported in literature[7].

Supplementary Table 3 | Scattering length densities of the components used in the SAXS analysis. Scattering length densities (SLDs) were calculated using the SasView SLD calculator[8] for an X-ray energy of 20 keV.

**material**	**density** (g/cm^3)	**SLD** (10^{-6} Å^{-2})
toluene	0.867	8.0
acetone	0.784	7.3
$PbBr_2$	6.66	46.4
$CsPbBr_3$	4.55	32
n-hexane	0.655	6.5

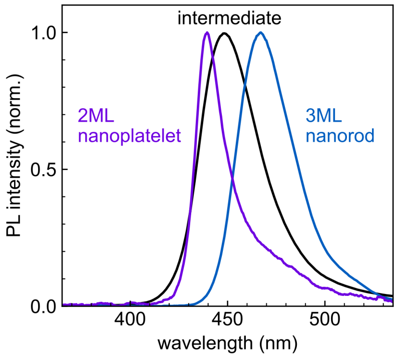

Supplementary Fig. 12 | Comparison of the PL of intermediate nanoclusters, 2ML nanoplatelets, and 3ML nanorods. 2 monolayer (ML) nanoplatelets show photoluminescence (PL) emission blueshifted compared to the intermediate nanoclusters. 3ML nanorods, on the other hand, show redshifted emission. PL intensities are extracted from in situ data. Therefore, the emission of 3ML nanorods appears broadened and slightly redshifted compared to purified samples, likely also due to the presence of few, thicker nanocrystals.

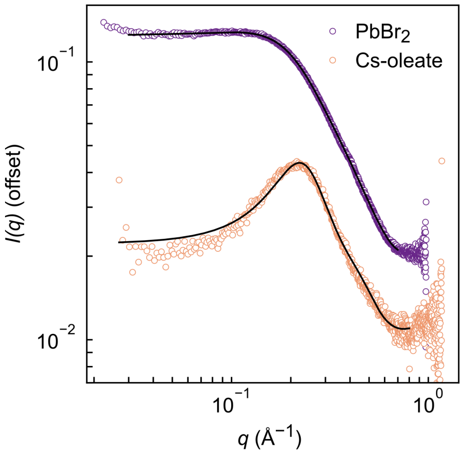

Supplementary Fig. 13 | SAXS analysis of the $PbBr_2$- and the Cs-oleate precursor. Solid lines are fits to the data using a model of core-shell spheres interacting by a hard sphere interaction. The precursors are described as micelles, i.e., cores of $PbBr_2$ or Cs surrounded by organic ligands. Fit parameters are given in Supplementary Table 4.

Supplementary Table 4 | SAXS fit parameters of the precursors. List of parameters obtained from the model fit to the small-angle X-ray scattering (SAXS) data of the $PbBr_2$- and the Cs-oleate precursor. Uncertainties are provided by SasView's Differential Evolution Adaptive Metropolis (DREAM) algorithm and correspond to 68% confidence intervals.

**parameter**	**$PbBr_2$**	**Cs-oleate**
scale (arb.u.)	0.239 ± 0.007	9.1 ± 0.6
background (arb.u.)	$(2.07 \pm 0.02) \cdot 10^{-2}$	$(5.5 \pm 0.7) \cdot 10^{-4}$
core diameter (nm)	1.19 ± 0.09	1.21 ± 0.02
shell thickness (nm)	0.820 ± 0.008	0.625 ± 0.009
core SLD (10^{-6}Å^{-2})	46.6 (fixed)	1 (fixed)
shell SLD (10^{-6}Å^{-2})	8.76 ± 0.03	0.017 ± 0.003
solvent SLD (10^{-6}Å^{-2})	8 (fixed)	0 (fixed)
hard sphere volume fraction (unitless)	0.0389 ± 0.0008	0.181 ± 0.003

Supplementary Table 5 | Number of precursor micelles involved in intermediate nanocluster nucleation. The micelle core diameter d was found by small-angle X-ray scattering (SAXS) analysis shown in Supplementary Fig. 13 and Supplementary Table 4. The intermediate nanocluster core volume was found by SAXS analysis shown in Supplementary Tables 6 and 7. The core volume was calculated via $V = \frac{4\pi}{3}\left(\frac{d}{2}\right)^3$, where d is the core diameter, $V = \frac{4\pi}{3}\frac{l}{2}\left(\frac{d}{2}\right)^2$ (ellipsoid), or $V = a \cdot b \cdot c$ (cuboid). The core was assumed to consist of either bulk $PbBr_2$ or bulk $CsPbBr_3$, i.e., the involved ligands were attributed fully to the shell of the micelle or nanocluster. The number of Pb per micelle or nanocluster was calculated as $N = \frac{V\rho}{M}N_A$, where N_A is Avogadro's number. Uncertainties were propagated based on the uncertainty of the volume.

	**$PbBr_2$**	**3ML nanorods initial**	**3ML nanorods final**	**2ML nanoplatelets initial**	**2ML nanoplatelets final**
core volume V (nm^3)	0.9 ± 0.2	8.4 ± 0.2	16.6 ± 0.1	7.0 ± 1.1	11.5 ± 0.2
mass density ρ (g/cm^3)	6.66	4.55	4.55	4.55	4.55
molar mass M (g/mol)	367.01	579.82	579.82	579.82	579.82
number of Pb per particle N	9.6 ± 2.2	40 ± 1	78 ± 1	33 ± 6	55 ± 2

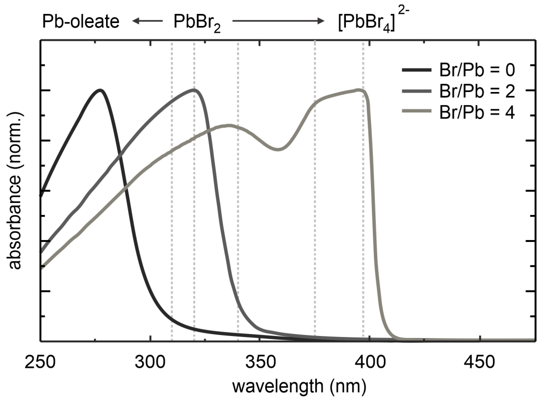

Supplementary Fig. 14 | Absorbance spectra of $PbBr_2$ precursor. Ex situ absorbance spectra of $PbBr_2$ precursor. Grey dotted lines mark typical absorption wavelengths of various Pb/Br-species observed in literature, i.e., 313 nm, 323 nm ($PbBr_2$ nanocrystals) 340 nm, 375 nm and 395 nm (1 monolayer (ML) nanosheets, $L_2[PbBr_4]$ with L denoting a surface coordination by an alkylamine ligand)[9,10]. The $PbBr_2$ precursor solution with oleylamine, oleic acid in toluene (Br/Pb = 2) absorbs strongly around 325 nm, which is similar to the absorption of $PbBr_2$ nanocrystals, supporting a model of ligand-stabilised $PbBr_2$ micelles. For comparison, a Pb-oleate precursor in oleic acid (Br/Pb = 0) absorbs below 300 nm while a $PbBr_2$ precursor solution with excess bromide (Br/Pb = 4) shows strong absorption up to 396 nm, hinting at the presence of 1ML $L_2[PbBr_4]$ nanosheets. The emerging shoulder around 375 nm has been observed as a prestage during the formation of 1ML $L_2[PbBr_4]$ nanosheets[10].

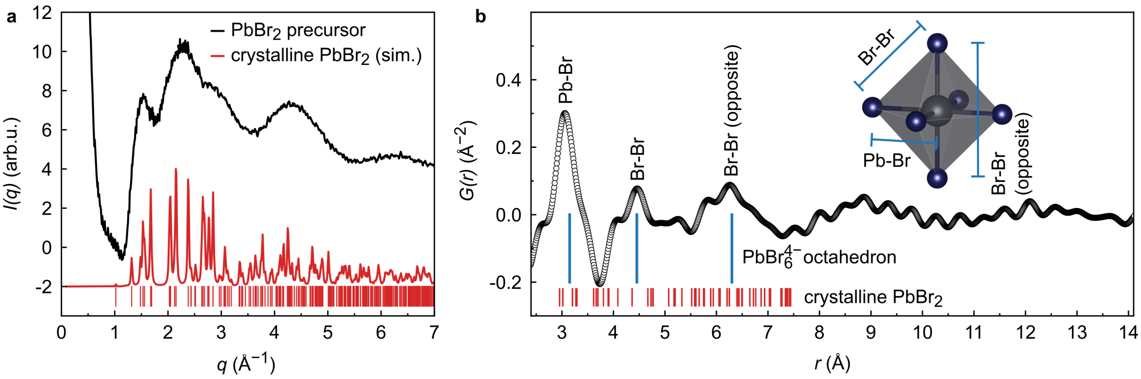

Supplementary Fig. 15 | WAXS and PDF analysis of the $PbBr_2$ precursor. **a**, In situ small- and wide-angle X-ray scattering (SAXS/WAXS) intensity of the $PbBr_2$ precursor (black line) and simulated intensity of crystalline $PbBr_2$ (Crystallography Open Database (COD) 1530324, red)[11,3]. Instead of sharp crystalline reflections the signal of the $PbBr_2$ precursor shows broad intensity oscillations extending to large q. This indicates that the precursor is either nanocrystalline with a very small crystallite size ($\leq$ 1.2 nm, the core diameter of the precursor micelles found by small-angle X-ray scattering (SAXS) analysis), or present as short-range ordered nanoclusters. **b**, Open dots: Reduced pair distribution function (PDF) $G(r)$ calculated from the background-subtracted WAXS/total scattering data of the $PbBr_2$ precursor shown in **a**, as detailed in Supplementary Note 2. Three major peaks at 3.06, 4.46, and 6.27 Å can be identified. These positions match the interatomic distances of Pb-Br, Br-Br, and Br-Br (opposite) inside a $PbBr_6{}^{4-}$ octahedron (inset), shown as blue lines. The interatomic distances for bulk crystalline $PbBr_2$ (COD 1530324, below 7.5 Å) are shown for comparison as red lines and do not match the observed distances. This indicates that the lead ion in the $PbBr_2$ precursor is likely six-fold coordinated by either Br or O/N, which belong to the oleic acid or oleylamine ligands. Such a coordination is similar to the solvent coordination of polyiodide plumbate complexes described in literature[12]. Interatomic distances were calculated using BondCalculator from the diffpy-cmi package[13]. Parameters for the PDF transformation are given in Supplementary Table 2. Due to the elevated solvent background in the in situ synthesis a rather small q_{max} of 14.3 Å^{-1} was chosen to reduce noise at low r while maintaining sharp peaks in the data. Above 7.5 Å such noise is visible as an oscillation in $G(r)$.

3.2 SAXS intensity model of the intermediate nanoclusters

A linear combination of the aforementioned $PbBr_2$ precursor intensity and the form factor of noninteracting compact particles was used as a model to analyse the change of SAXS intensity after Cs-oleate injection. The added Cs-oleate was not included as a separate component in the model to avoid overfitting. Different shapes of the compact particles were tested to reduce bias based on the choice of model: spheres, cubes, ellipsoids, cylinders and cuboids. Models of non-compact particles, i.e., rods or platelets extended beyond the resolution limit $\frac{2\pi}{q_{min}} \approx 28$ nm of the experiment in at least one direction, do not level off to a constant intensity at low q and did therefore not provide good agreement with the data. The differences between the different modelled particle shapes are described below.

During fitting, the Differential Evolution Adaptive Metropolis (DREAM) algorithm was used to minimise χ^2

$$\chi^2 = \frac{1}{N_{\text{pts}} - N_{\text{par}}} \sum_{N_{\text{pts}}} \left(\frac{I_{\text{data}} - I_{\text{model}}}{dI_{\text{data}}} \right)^2 \tag{S4}$$

N_{pts} is the number of data points in the fit range. N_{par} is the number of free parameters. I_{data} are the SAXS intensities with respective uncertainties dI_{data}, and I_{model} is the model function evaluated on the data points. Lower values of χ^2 indicate a better fit, but potential overfitting by correlated parameters and plausibility of the minimal parameters must be assessed manually. This procedure is outlined below.

In the case of spheres, fits were attempted with monodisperse spheres, polydisperse spheres with a unimodal Gaussian distribution of diameter, and with two possible diameters. Exemplary results are shown in Supplementary Fig. 16 and Supplementary Tables 6 and 7 for a synthesis of 3ML nanorods and a synthesis of 2ML nanoplatelets, for the last frame before antisolvent injection.

Next, a simple model of particle anisotropy was tested by using ellipsoids or cylinders, both of which have two size parameters, diameter d and length l. First, fits with monodisperse, anisotropic particles were attempted. Then, Gaussian polydispersity (PD) for both size parameters was allowed. Results are shown in Supplementary Fig. 16. In all fits, the scales of the precursor and the growing particles, the constant background, and the size parameters were allowed to vary. This results in 4 (monodisperse spheres), 5 (polydisperse, unimodal spheres; two diameters of spheres; monodisperse ellipsoids; monodisperse cylinders) or 7 free parameters (polydisperse ellipsoids; polydisperse cylinders).

Also fits with cubic and cuboidal models were carried out due to the rectangular appearance of the perovskite cores of nanorods and nanoplatelets in TEM images. These models have 4 or 5 free parameters for monodisperse and polydisperse particles, respectively. In the case of cuboids (also called rectangular prisms or rectangular parallelepipeds),

either two sides were constrained to have the same length, or all three sides were refined separately, leading to 5 or 6 free parameters, respectively. Due to the numerical integration involved in the orientational averaging of these models computation time is much longer compared to the other models. Therefore a screening of polydispersity was only carried out on selected datasets but did not lead to a significant further improvement.

All spherical models did not provide good agreement with the data, as emphasised by the plot of the weighted residual, red and brown curves in Supplementary Fig. 16. The remaining oscillations in the residual indicate a poor fit. Furthermore, the corresponding size parameters were not found to be plausible. Either the diameter was too small (< 1 nm mean diameter for polydisperse spheres, on the order of single $PbBr_6$ octahedra) or too large (3 to 4 nm for monodisperse spheres, 2.1 to 4.1 nm for two populations of spheres), as no redshifted photoluminescence emission, characteristic of 3 to 4 nm sized perovskite nanocrystals, was observed. Furthermore, the temporal evolution of mean diameter and polydispersity for the model of polydisperse spheres, shown in Supplementary Fig. 17 a-c exhibits constantly too small mean diameter and too large polydispersity, which leads to rejection of the model. A model of a bimodal (two-Gaussian) distribution of diameter was found unstable due to the large number of free parameters. The cubic models, with and without polydispersity, also showed poor agreement with the data and strongly oscillating residuals (orange curves in Supplementary Fig. 16).

The simplest anisotropic models (monodisperse ellipsoids and monodisperse cylinders) provided good agreement with the data, as indicated by low values of χ^2 at relatively low complexity (green and blue curves in Supplementary Fig. 16). Allowing polydispersity further improved the fit, as indicated by the flatter residuals, but the mean values of the small particle dimensions are unrealistically small, and the associated polydispersity is very high, especially along the short dimension.

Cuboid models also describe the data well (purple curves in Supplementary Fig. 16), and the obtained dimensions are very close to the dimensions from the ellipsoid and cylinder models. Allowing three lengths of the cuboid to be refined slightly improved the fit. However, no evidence for two distinct short sides of the intermediate nanoclusters was found by TEM or PL analysis (Fig. 3 f,g and Supplementary Fig. 11), so the less complex model of a cuboid with two identical sides is preferred. Ellipsoid, cylinder, and cuboid models all yield similar sizes for the short sides of the intermediate nanoclusters, the smallest being the cuboid length and the largest the ellipsoid diameter. This is expected as the scattering contrast of the perovskite nanoclusters to the solvent has likely no sharp boundary due to the presence of a ligand shell with intermediate contrast. Therefore a cuboid model yields size parameters closer to the perovskite core size whereas an ellipsoid model is a better description for the nanocluster including the ligand shell. As a result, the ellipsoidal model, representing a slightly rounded particle, is preferred for a description of the particles when covered with ligands and in a dispersed state. A cuboid model might be more appropriate for particles stripped of ligands or in direct contact with each other. Finally, the ellipsoid model was chosen for further analysis due to faster computation.

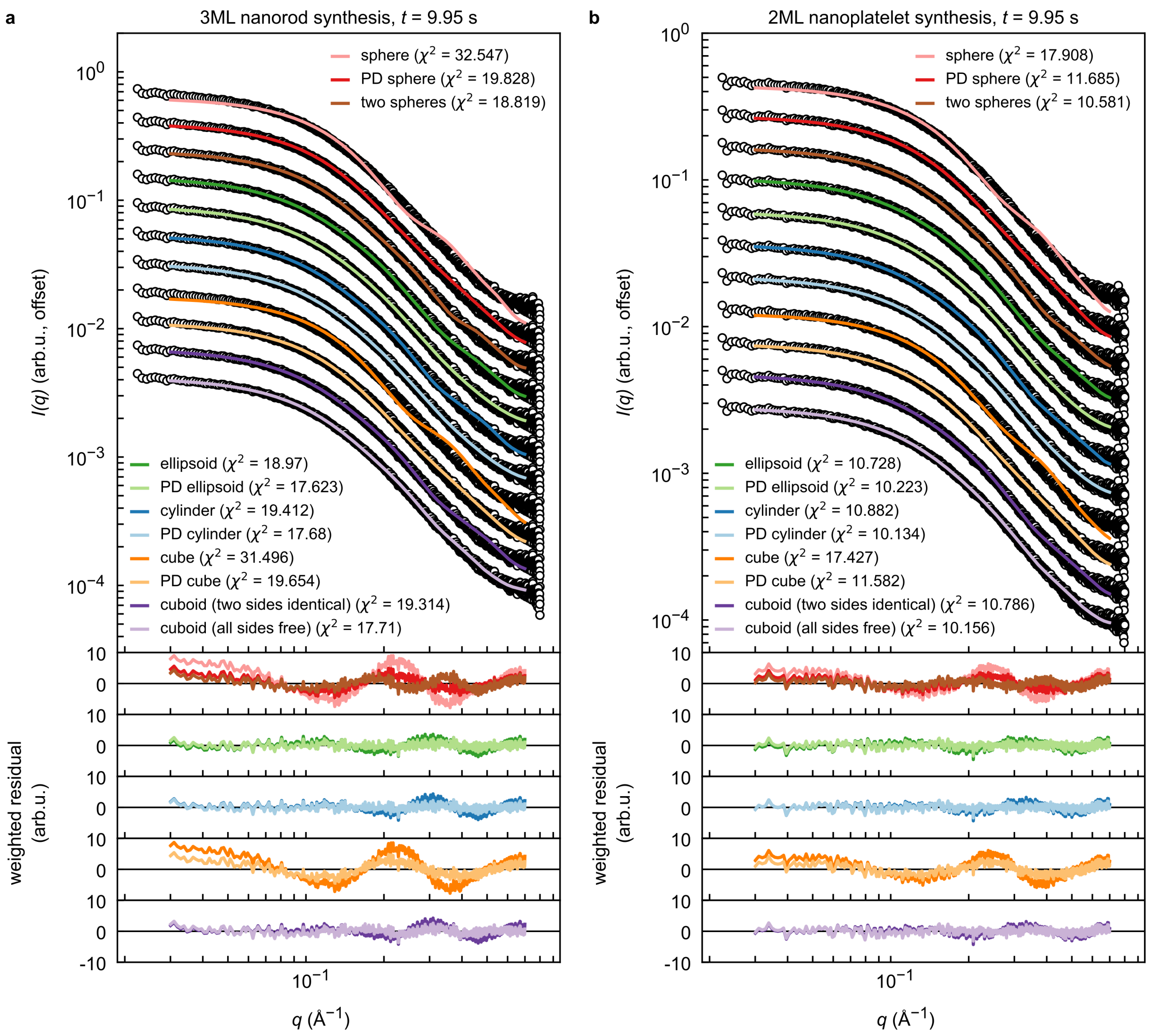

Supplementary Fig. 16 | Comparison of different shape models of the intermediate nanoclusters. **a**, Fit of the last frame before antisolvent injection for the synthesis of 3 monolayer (ML) nanorods. "PD" indicates a Gaussian size polydispersity in the model. Sphere models (red and brown curves) provide a poor fit; note the oscillating residuals. Cube models (orange curves) also show large and oscillating residuals. Anisotropic models (ellipsoid, cylinder, cuboid) provide good fits, showing, on average, lower values of χ^2 and flat residuals over the fitted range (green, blue, and purple curves). All anisotropic models result in prolate particles. A slight improvement of the fit can be achieved by allowing polydispersity (for ellipsoid and cylinder models) or allowing three different dimensions in the cuboid model compared to a model in which two sides are constrained equally. **b**, Fit of the last frame before antisolvent injection for the synthesis of 2ML nanoplatelets. Again, the sphere and cube models do not agree well with the data, whereas ellipsoid, cylinder and cuboid models provide satisfactory fits, indicating that nanocluster anisotropy is key to obtaining a good fit to the data.

Supplementary Table 6 | SAXS fit parameters for different particle shape models for 3ML nanorod synthesis. Uncertainties are provided by the Differential Evolution Adaptive Metropolis (DREAM) algorithm of SasView and correspond to 68 % confidence intervals. Note that the "two spheres", "PD cube", and "cuboid two sides identical" models are not implemented on an absolute intensity scale and, therefore, provide different scale parameters from the other models. Relative polydispersity (rel. PD) is defined as the ratio of standard deviation and mean $\frac{\sigma_l}{l}$ or $\frac{\sigma_d}{d}$ of a Gaussian distribution of sizes.

	**sphere**	**PD sphere**	**two spheres**
precursor scale (arb.u.)	0.2506 ± 0.0008	0.1912 ± 0.0009	0.5468 ± 0.0005
nanocluster scale (arb.u.)	$(4.34 \pm 0.02) \cdot 10^{-4}$	$(6.72 \pm 0.03) \cdot 10^{-4}$	$(1.84 \pm 0.03) \cdot 10^{6}$
nanocluster scale 2 (arb.u.)	-	-	$(4.70 \pm 0.04) \cdot 10^{6}$
background (arb.u.)	$(1.045 \pm 0.007) \cdot 10^{-2}$	$(1.243 \pm 0.007) \cdot 10^{-2}$	$(1.327 \pm 0.008) \cdot 10^{-2}$
nanocluster diameter (nm) (rel. PD in brackets)	3.395 ± 0.005	0.67 ± 0.02 (2.00 ± 0.04)	4.13 ± 0.02
nanocluster diameter 2 (nm)	-	-	2.38 ± 0.02
χ^2	32.547	19.828	18.819
	**ellipsoid**	**PD ellipsoid**	
precursor scale (arb.u.)	0.185 ± 0.002	0.112 ± 0.003	
nanocluster scale (arb.u.)	$(6.405 \pm 0.05) \cdot 10^{-4}$	$(1.134 \pm 0.002) \cdot 10^{-2}$	
background (arb.u.)	$(1.322 \pm 0.008) \cdot 10^{-2}$	$(1.393 \pm 0.008) \cdot 10^{-2}$	
nanocluster length l (nm) (rel. PD in brackets)	6.14 ± 0.03	5.71 ± 0.04 (0.08 ± 0.04)	
nanocluster diameter d (nm) (rel. PD in brackets)	2.269 ± 0.008	0.49 ± 0.01 (1.87 ± 0.05)	
χ^2	18.97	17.623	
	**cylinder**	**PD cylinder**	
precursor scale (arb.u.)	0.191 ± 0.002	0.121 ± 0.002	
nanocluster scale (arb.u.)	$(6.24 \pm 0.04) \cdot 10^{-4}$	$(1.07 \pm 0.10) \cdot 10^{-4}$	
background (arb.u.)	$(1.300 \pm 0.008) \cdot 10^{-2}$	$(1.393 \pm 0.008) \cdot 10^{-2}$	
nanocluster length l (nm) (rel. PD in brackets)	4.45 ± 0.02	4.01 ± 0.04 (0.19 ± 0.02)	
nanocluster diameter d (nm) (rel. PD in brackets)	2.180 ± 0.007	0.49 ± 0.02 (1.86 ± 0.07)	
χ^2	19.412	17.68	
	**cube**	**PD cube**	
precursor scale (arb.u.)	0.729 ± 0.002	0.551 ± 0.003	
nanocluster scale (arb.u.)	263 ± 2	$(2.54 \pm 0.03) \cdot 10^{3}$	
background (arb.u.)	$(1.052 \pm 0.007) \cdot 10^{-2}$	$(1.24 \pm 0.008) \cdot 10^{-2}$	
nanocluster size (nm) (rel. PD in brackets)	2.645 ± 0.004	0.548 ± 0.008 (1.89 ± 0.03)	
χ^2	31.496	19.654	
	**cuboid two sides identical**	**cuboid all sides free**	
precursor scale (arb.u.)	0.565 ± 0.004	0.132 ± 0.005	
nanocluster scale (arb.u.)	414 ± 5	$(9.2 \pm 0.3) \cdot 10^{-4}$	
background (arb.u.)	$(1.295 \pm 0.008) \cdot 10^{-2}$	$(1.44 \pm 0.02) \cdot 10^{-2}$	
nanocluster length a (nm)	1.890 ± 0.006	4.23 ± 0.02	
nanocluster length b (nm)	1.890 ± 0.006	2.31 ± 0.02	
nanocluster length c (nm)	4.44 ± 0.02	1.19 ± 0.03	
χ^2	19.314	17.71	

Supplementary Table 7 | SAXS fit parameters for different particle shape models for 2ML nanoplatelet synthesis. Uncertainties are provided by the Differential Evolution Adaptive Metropolis (DREAM) algorithm of SasView and correspond to 68 % confidence intervals. Note that the "two spheres", "PD cube", and "cuboid two sides identical" models are not implemented on an absolute intensity scale and, therefore, provide different scale parameters from the other models. Relative polydispersity (rel. PD) is defined as the ratio of standard deviation and mean $\frac{\sigma_l}{l}$ or $\frac{\sigma_d}{d}$ of a Gaussian distribution of sizes.

	**sphere**	**PD sphere**	**two spheres**
precursor scale (arb.u.)	0.2651 ± 0.0008	0.215 ± 0.002	0.581 ± 0.006
nanocluster scale (arb.u.)	$(3.33 \pm 0.02) \cdot 10^{-4}$	$(5.46 \pm 0.04) \cdot 10^{-4}$	$(4.21 \pm 0.07) \cdot 10^{6}$
nanocluster scale 2 (arb.u.)	-	-	$(1.50 \pm 0.04) \cdot 10^{6}$
background (arb.u.)	$(1.213 \pm 0.007) \cdot 10^{-2}$	$(1.3616 \pm 0.008) \cdot 10^{-2}$	$(1.4534 \pm 0.009) \cdot 10^{-2}$
nanocluster diameter (nm) (rel. PD in brackets)	3.163 ± 0.006	0.61 ± 0.02 (2.00 ± 0.06)	2.13 ± 0.02
nanocluster diameter 2 (nm)	-	-	3.78 ± 0.02
χ^2	17.908	11.685	10.581
	**ellipsoid**	**PD ellipsoid**	
precursor scale (arb.u.)	0.198 ± 0.002	0.117 ± 0.005	
nanocluster scale (arb.u.)	$(5.5667 \pm 0.008) \cdot 10^{-4}$	$(1.18 \pm 0.04) \cdot 10^{-3}$	
background (arb.u.)	$(1.4629 \pm 0.009) \cdot 10^{-2}$	$(0.0150 \pm 0.009) \cdot 10^{-2}$	
nanocluster length l (nm) (rel. PD in brackets)	5.56 ± 0.03	5.32 ± 0.03 (0.00 ± 0.03)	
nanocluster diameter d (nm) (rel. PD in brackets)	2.00 ± 0.02	0.60 ± 0.02 (1.15 ± 0.05)	
χ^2	10.728	10.223	
	**cylinder**	**PD cylinder**	
precursor scale (arb.u.)	0.205 ± 0.002	0.129 ± 0.004	
nanocluster scale (arb.u.)	$(5.40 \pm 0.06) \cdot 10^{-4}$	$(1.07 \pm 0.03) \cdot 10^{-3}$	
background (arb.u.)	$(1.439 \pm 0.009) \cdot 10^{-2}$	$(1.497 \pm 0.009) \cdot 10^{-2}$	
nanocluster length l (nm) (rel. PD in brackets)	4.04 ± 0.02	3.65 ± 0.03 (0.16 ± 0.02)	
nanocluster diameter d (nm) (rel. PD in brackets)	1.94 ± 0.01	0.56 ± 0.02 (1.27 ± 0.05)	
χ^2	10.882	10.134	
	**cube**	**PD cube**	
precursor scale (arb.u.)	0.761 ± 0.003	0.617 ± 0.003	
nanocluster scale (arb.u.)	250 ± 3	$(2.46 \pm 0.04) \cdot 10^{3}$	
background (arb.u.)	$(1.217 \pm 0.008) \cdot 10^{-2}$	$(1.356 \pm 0.008) \cdot 10^{-2}$	
nanocluster size (nm) (rel. PD in brackets)	2.463 ± 0.005	0.644 ± 0.008 (1.41 ± 0.02)	
χ^2	17.427	11.582	
	**cuboid two sides identical**	**cuboid all sides free**	
precursor scale (arb.u.)	0.595 ± 0.005	0.146 ± 0.006	
nanocluster scale (arb.u.)	501 ± 10	$(8.8 \pm 0.4) \cdot 10^{-4}$	
background (arb.u.)	$(1.436 \pm 0.009) \cdot 10^{-2}$	$(1.54 \pm 0.01) \cdot 10^{-2}$	
nanocluster length a (nm)	1.681 ± 0.009	3.82 ± 0.02	
nanocluster length b (nm)	1.681 ± 0.009	1.04 ± 0.03	
nanocluster length c (nm)	4.03 ± 0.02	1.96 ± 0.02	
χ^2	10.786	10.156	

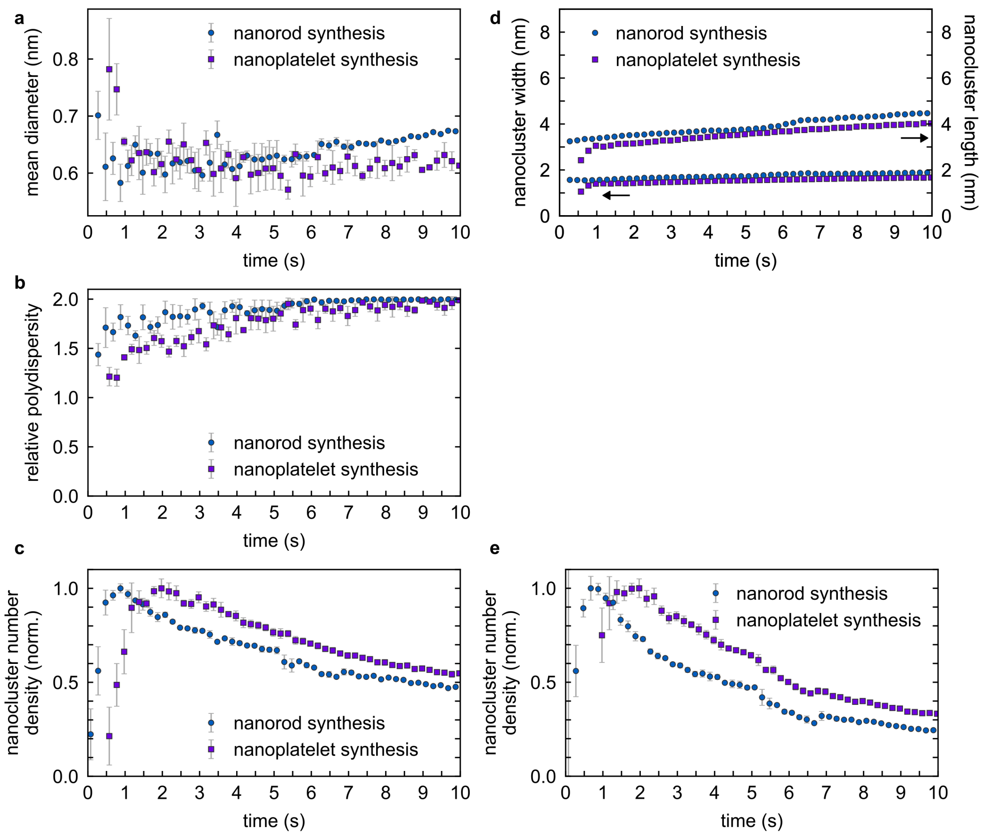

Supplementary Fig. 17 | SAXS fit results using a model of polydisperse spheres, or cuboids with two identical sides. **a**, Mean nanocluster diameter for nanorod and nanoplatelet synthesis, respectively, obtained using a model fit with polydisperse spheres as a model for the intermediate nanoclusters. The mean diameter is very small ($< 1\,\mathrm{nm}$), which is not plausible considering the crystallite size obtained from wide-angle X-ray scattering (WAXS) and the photoluminescence (PL) emission observed from these nanoclusters. **b**, Corresponding relative polydispersity from the fit. The values are very large and reach the upper limit of 2 imposed by the fit. This is in contradiction to the relatively narrow linewidth of the PL emission. **c**, Mean nanocluster number density obtained from the fit. Overall, the size parameters obtained from this model contradict the observations from the simultaneously recorded WAXS and PL data. Therefore, the model of polydisperse spheres was rejected. **d**, Nanocluster width and length for nanorod and nanoplatelet synthesis, respectively, obtained using a model fit with a cuboid model. Two sides are constrained to be equal. The resulting parameters correspond to prolate nanoclusters (width $a = b <$ length c) for all times and are in agreement with the results of the ellipsoid model shown in the main text. No polydispersity had to be assumed to fit the data. **e**, Corresponding mean nanocluster number density obtained from the fit. Error bars correspond to the standard deviation of four consecutive fit results.

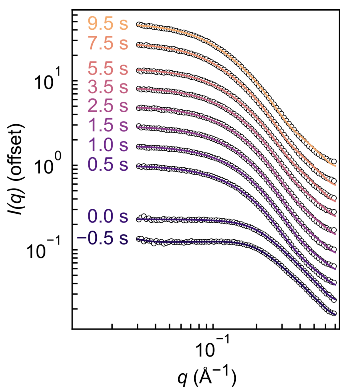

Supplementary Fig. 18 | SAXS analysis of intermediate nanocluster formation for a 3ML nanorod synthesis. Small-angle X-ray scattering (SAXS) intensities after Cs-oleate injection are shown as a function of scattering vector q and time. Solid lines are fits to the data. The nanocluster dimensions obtained from the fits are shown in Fig. 3.

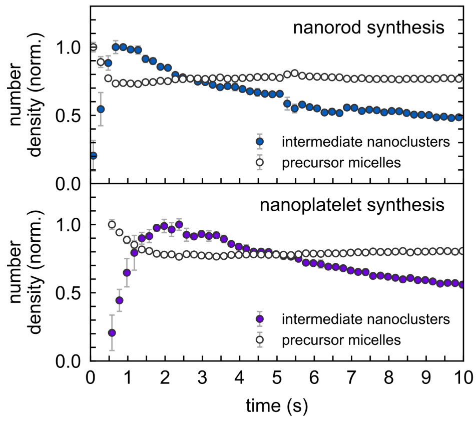

Supplementary Fig. 19 | Evolution of number densities of precursor micelles and intermediate nanoclusters. Number density of $PbBr_2$ precursor micelles and intermediate nanoclusters as a function of time after Cs-oleate injection ($t = 0$ s), obtained from small-angle X-ray scattering (SAXS) analysis. The initial drop of number density of precursor micelles coincides in time with the rapid nucleation of intermediate nanoclusters. The following slight increase is likely within the experimental uncertainty, to which the uncertainties of the fitted scale factors and of the precursor micelle core volume contribute. It should be noted that the number density of precursor micelles only decays to to 75-80% of the initial value. This indicates that only 20-25% of the $PbBr_2$ precursor material is consumed in the initial nucleation, a process which is limited by the availability of Cs-oleate precursor. Error bars correspond to the standard deviation of four consecutive fit results.

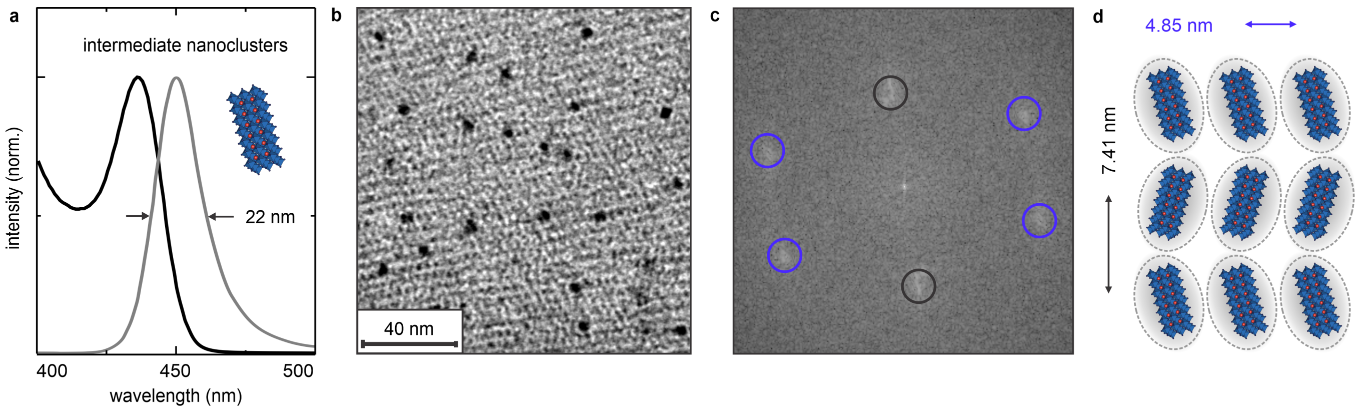

Supplementary Fig. 20 | Optical characterisation and TEM imaging of a precursor mixture containing perovskite intermediate nanoclusters. **a**, Absorbance and photoluminescence (PL) spectra with an excitonic absorption peak at 437 nm and a blue emission at 450 nm, full width at half maximum (FWHM) = 22 nm. The photoluminescence profile is identical to the in situ PL observed after precursor mixing and before antisolvent injection and, therefore, assumed to stem from the prolate, crystalline intermediate nanoclusters. **b**, Lower resolution transmission electron microscopy (TEM) image of a crude precursor mix, showing an ordered assembly of prolate particles in side-on view. The larger high contrast particles are likely Pb^0, which formed through the reduction of excess $PbBr_2$ precursor material by the electron beam[14]. **c**, Fast Fourier transform (FFT) of TEM image in **b**. Particle spacing in different directions is marked in blue $(4.85\,\text{nm})^{-1}$ and black $(7.41\,\text{nm})^{-1}$. These values are in good agreement with the final dimensions of intermediate nanoclusters obtained from fitting in situ small-angle X-ray scattering (SAXS) data, including a ligand double layer of 3 nm between individual clusters. **d**, Scheme of possible arrangement of intermediate nanoclusters derived from the FFT in **c**.

Supplementary Table 8 | Critical synthesis parameters used in the in situ SAXS/WAXS/PL experiments.

	**3ML nanorods**	**2ML nanoplatelets**
load $PbBr_2$ precursor	300 µL	400 µL
start SAXS, WAXS, and PL measurement		
inject Cs-oleate	30 µL	20 µL
wait and stir	10 s	10 s
inject acetone antisolvent	400 µL	267 µL
wait and stir	until $\leq$ 480 s total duration	
stop SAXS, WAXS, and PL measurement		

3.3 Analysis of the antisolvent-induced mesophase

Individual peaks in the SAXS data were fitted to analyse the structure of the antisolvent-induced mesophase using a Pseudo-Voigt function on a background as described below. The background was either modelled as linear or as a Guinier law, which is a simplified expression of the small-angle scattering intensity of compact objects with a radius of gyration R_g [15].

$$bkg_{linear}(q) = m \cdot q + t \tag{S5}$$

$$bkg_{Guinier}(q) = scale_{Guinier}\ \mathrm{e}^{-\frac{1}{3}(qR_g)^2} \tag{S6}$$

The model was implemented using the lmfit package for python [16]. Where appropriate, a chain fit procedure was used, going backwards in time and using the refined parameter values as the starting values for the next frame. Peak position, height, width, Lorentz/Gauss ratio and two to four background parameters were refined (6 to 8 free parameters). The fit results for the (1 0 0) and (0 0 1) reflections of a 3ML nanorod-forming mixture and a 2ML nanoplatelet-forming mixture are shown in Supplementary Figs. 21 and 22.

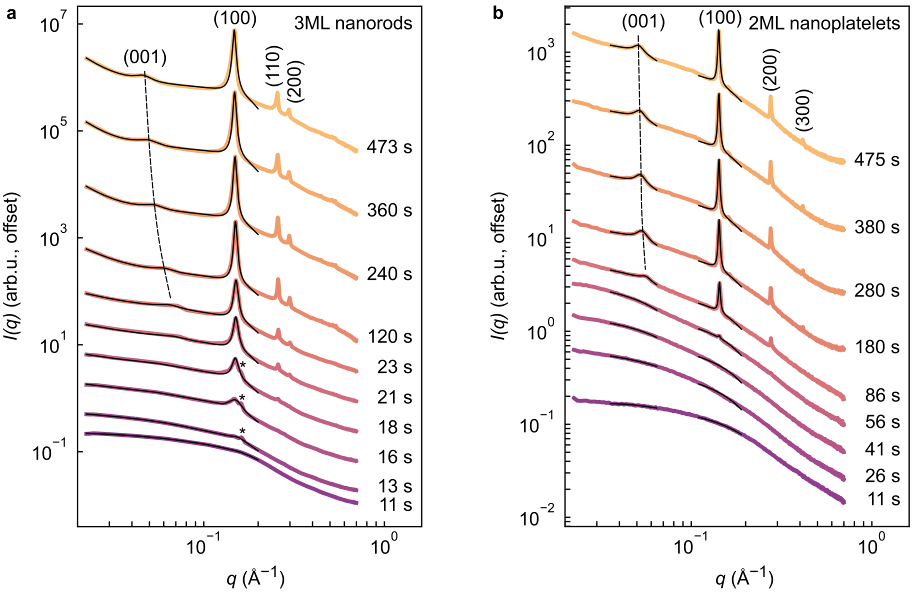

Supplementary Fig. 21 | SAXS data after antisolvent injection. a Selected in situ small-angle X-ray scattering (SAXS) intensities of a 3 monolayer (ML) nanorod synthesis. t = 0 s is the time of Cs-oleate injection, t = 10 s is the time of acetone injection. Mesophase formation starts immediately after acetone injection. Between 13 and 18 s an additional peak at larger q than the (1 0 0) reflection of the hexagonal mesophase is observed (highlighted by an asterisk *). This could originate from a mesophase consisting of ligands and smaller clusters than the intermediate nanoclusters, e.g., residual $PbBr_2$ remaining in the reaction mixture. After 21 s only the mesophase containing intermediate nanoclusters, i.e., growing nanorods, is observed. Note the pronounced shift of the low angle (0 0 1) reflection, which signifies the growth of the nanorods along the nanorod axis. **b** Selected in situ SAXS intensities of a 2ML nanoplatelet synthesis. Lamellar mesophase formation is delayed by approximately 60 s. (0 0 1) and (1 0 0) reflections were analysed separately and correspond to the stacking of nanoplatelets along the long and short axis, respectively.

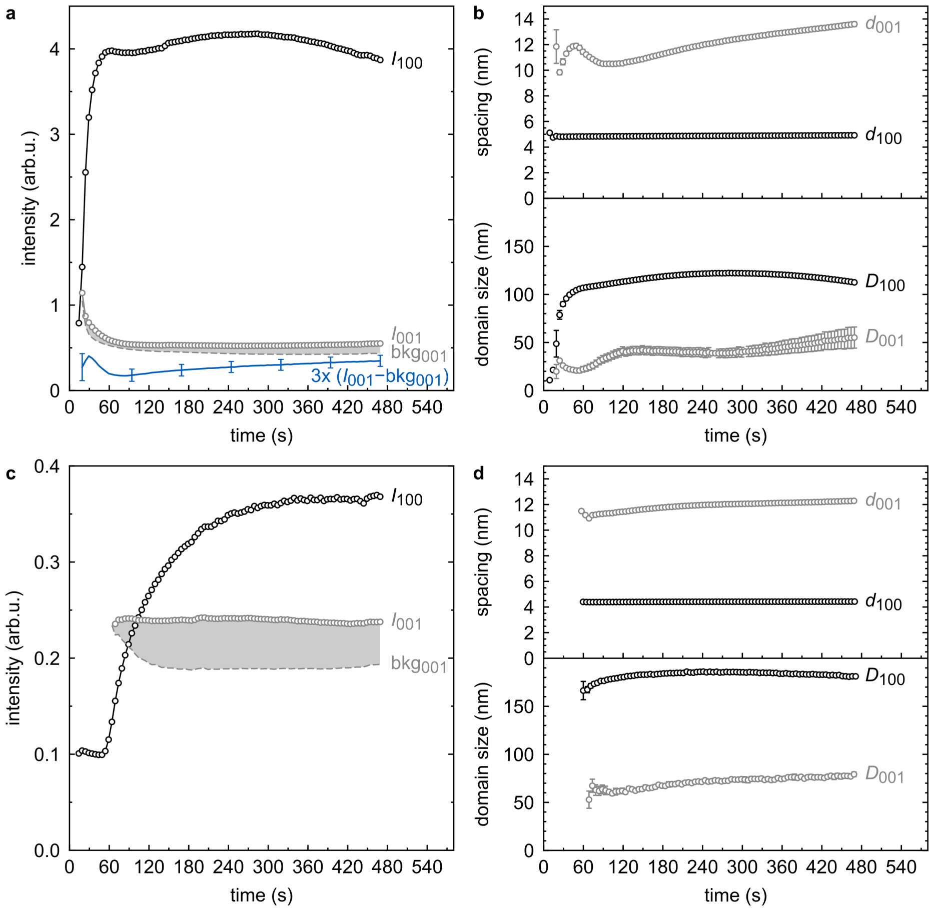

Supplementary Fig. 22 | SAXS analysis of the mesophase reflections. **a**, Intensity at the mesophase peak positions for a 3 monolayer (ML) nanorod synthesis as a function of time. Intensities are obtained from the fits shown in Supplementary Fig. 21. For the (0 0 1) reflection the evolution of the background (bkg) at the peak position is additionally shown. The blue line shows the resulting (0 0 1) peak intensity and the associated uncertainty as error bars, assuming 5% uncertainty of the background level. **b**, Lattice spacings d and domain sizes D obtained from fits of the (1 0 0) and (0 0 1) peaks in **a**. **c**, Intensity at the mesophase peak positions for a 2ML nanoplatelet synthesis. **d**, Lattice spacings d and domain sizes D obtained from fits of the (1 0 0) and (0 0 1) peaks in **c**. Lattice spacings are calculated as $\frac{4\pi}{\sqrt{3}q_{(100)}}$, $\frac{2\pi}{q_{(001)}}$ for the hexagonal phase and $\frac{2\pi}{q_{(100)}}$, $\frac{2\pi}{q_{(001)}}$ for the lamellar phase. Domain sizes are calculated as $\frac{2\pi}{\Delta q}$. Δq is the full width at half maximum of the fitted peak, assuming no additional broadening due to instrumental effects or lattice strain. Dots with error bars in **b** and **d** show the mean values and standard deviations of ten successive fit results.

4 Variation of precursor concentration and antisolvent volume

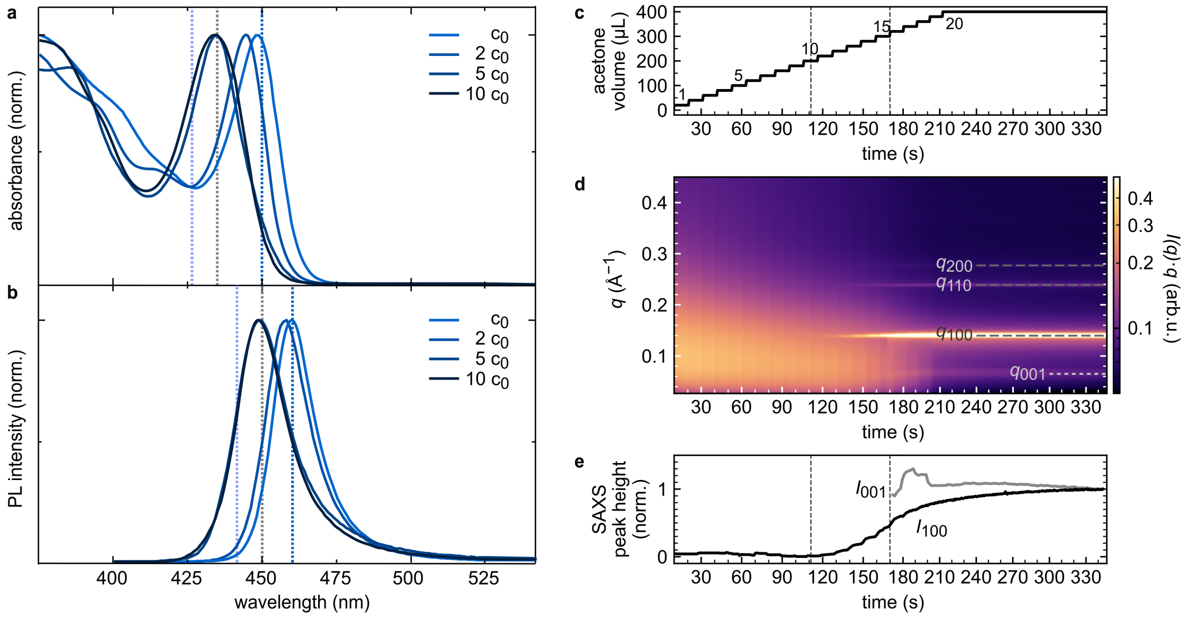

Supplementary Fig. 23 | Comparison of synthesis products at different precursor concentrations and antisolvent volumes. **a**, Absorbance and **b**, photoluminescence (PL) of purified samples from synthesis at different precursor concentrations, leaving the volume of antisolvent constant, given as multiples of the standard concentration c_0. Concentrations close to the standard concentration (c_0 and 2 c_0) lead to the formation of a mesophase, which stabilises the intermediate nanoclusters sufficiently well against dissolution. Neighbouring intermediate nanoclusters in the mesophase can, however, still fuse to nanorods, and therefore, the characteristic PL emission of 3 monolayer (ML) nanorods is obtained for these concentrations. For higher concentrations (5 c_0 and 10 c_0), intermediate nanoclusters are again assembled into the mesophase. However, the stabilising effect exceeds the tendency for fusion of intermediate nanoclusters, which stabilises emission at 450 nm. Vertical dotted lines indicate the characteristic absorption and emission features of 2ML nanoplatelets (428 nm, 434 nm), intermediate nanoclusters (437 nm, 450 nm), and 3ML nanorods (448 nm, 460 nm), respectively. **c-e**, Study of mesophase formation by in situ small-angle X-ray scattering (SAXS). **c** Acetone was added as an antisolvent in 20 steps of 20 µL to a highly concentrated reaction mixture at 5 c_0 to find the minimal volume necessary for mesophase formation. After 10 injections (200 µL), the Bragg reflections of the hexagonal mesophase with a spacing of (5.23 ± 0.03) nm are first observed in the SAXS intensity (vertical dashed line). After 16 injections (320 µL), another reflection at lower q indicates the stacking of intermediate nanoclusters along their long axis at a final spacing of 9.7 nm. Compared to the regular 3ML nanorod synthesis shown in Fig. 4 and Supplementary Figs. 21 and 22, no further increase of this spacing is observed. This indicates that high precursor concentration, and therefore a higher concentration of intermediate nanoclusters, promotes mesophase formation, but the lower relative fraction of antisolvent does not lead to the necessary weakening of the ligand shell, which would induce fusion of intermediate nanoclusters.

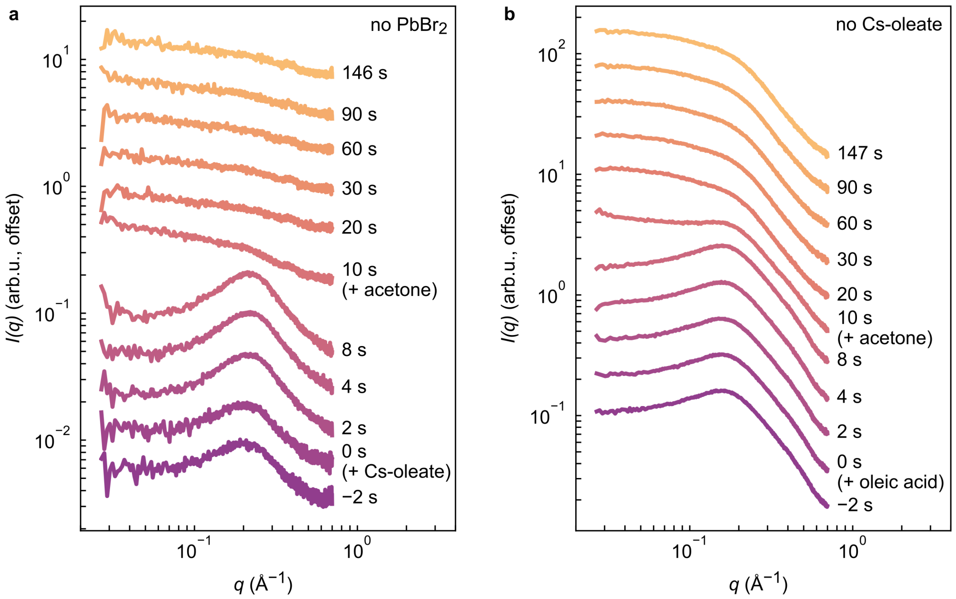

Supplementary Fig. 24 | SAXS data of syntheses without $PbBr_2$ or Cs-oleate. **a**, Selected in situ small-angle X-ray scattering (SAXS) intensities of a synthesis in which $PbBr_2$ was omitted from the first precursor. No perovskite nanoclusters or nanocrystals can be formed in the absence of $PbBr_2$. This results in a much lower SAXS intensity compared to a regular synthesis, visible as a higher noise level. Before Cs-oleate injection, a faint signal of ligand micelles can be seen, which is slightly increased by the addition of the micellar Cs-oleate precursor. Acetone leads to a dilution of the mixture, resulting in a disappearance of the structure factor peak at $q = 0.22\,\text{Å}^{-1}$ and instead a smoothly decaying intensity indicative of the micelle form factor. No Bragg reflections of a mesophase are observed. **b**, Selected in situ SAXS intensities of a synthesis in which the Cs-oleate precursor was replaced by the corresponding volume of oleic acid. No perovskite nanoclusters or nanocrystals can be formed in the absence of Cs. Until acetone injection, the signal of $PbBr_2$ precursor micelles is dominant and not visibly affected by the additional ligands. Acetone injection leads to a dilution of the $PbBr_2$ precursor micelles. The structure factor peak at $q = 0.17\,\text{Å}^{-1}$ is weakened compared to the signal of the form factor. Again, no Bragg reflections of a mesophase are observed.

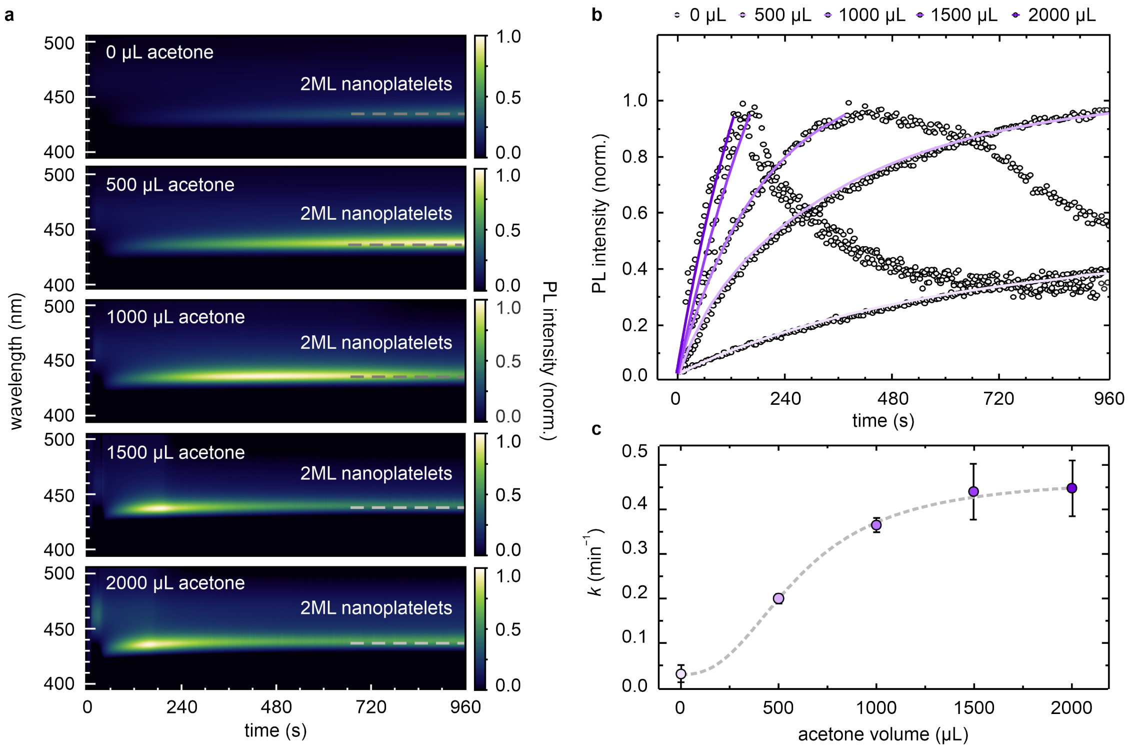

Supplementary Fig. 25 | In situ PL of 2ML nanoplatelet syntheses with varying volumes of acetone antisolvent. **a**, In situ photoluminescence (PL) of 2 monolayer (ML) nanoplatelet syntheses at standard concentration c_0 with varying volumes of acetone injected at $t = 10\,\mathrm{s}$. **b**, Extracted PL intensities from **a**, fitted with an exponential association function (purple lines) from $t = 0\,\mathrm{s}$ to the point at which the maximum PL intensity is reached. In each case, monodisperse 2ML nanoplatelets form, but the addition of polar antisolvents, like acetone, notably accelerates their formation. The PL intensity decrease in syntheses with ≥ 1000 µL of acetone is attributed to increased reabsorption and scattering due to the formation and precipitation of the lamellar stacked mesophase of 2ML nanoplatelets. At standard concentration c_0, this also occurs in syntheses with < 1000 µL acetone after longer reaction times $t > 960\,\mathrm{s}$. **c**, Rate constants for 2ML nanoplatelet growth extracted from the exponential association fits (Supplementary Table 9) of in situ PL in **b**, showing an acceleration from $k = 0.03\,\mathrm{min}^{-1}$ to $k = 0.44\,\mathrm{min}^{-1}$ with injection of acetone to the precursor mixture. Error bars indicate the respective fitting error.

Extracted and normalized PL intensities of 2ML nanoplatelet syntheses at standard precursor concentration and varying volumes of acetone as antisolvent were fitted with an exponential association function with amplitude A and rate k.

$$I(t) = A \cdot (1 - \mathrm{e}^{-kt}) \tag{S7}$$

Supplementary Table 9 | Exponential association fit parameters for growth of 2ML nanoplatelets at standard concentration with different volumes of acetone.

acetone volume (µL)	A	k (min^{-1})	R^2
0	0.998 ± 0.002	0.0272 ± 0.020	0.9909
500	0.997 ± 0.003	0.1972 ± 0.012	0.9914
1000	1.065 ± 0.007	0.3641 ± 0.018	0.9931
1500	2.285 ± 0.011	0.4349 ± 0.070	0.9931
2000	2.299 ± 0.011	0.4414 ± 0.072	0.9930

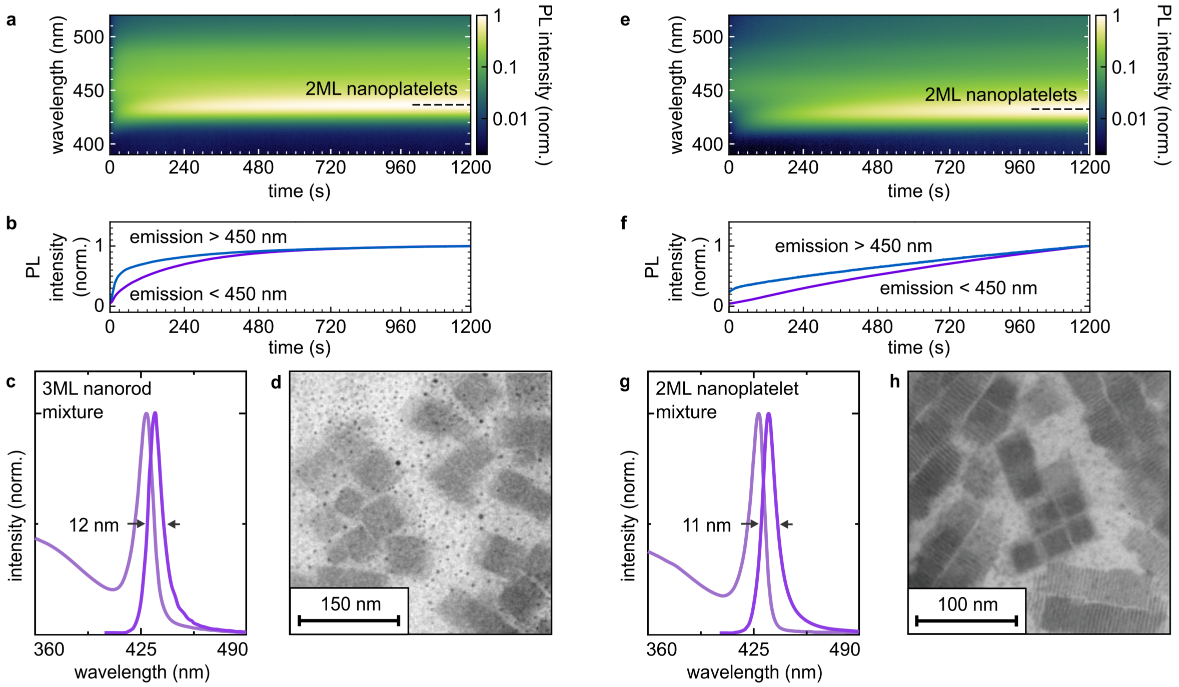

Supplementary Fig. 26 | In situ PL and ex situ optical and morphological characterisation of syntheses without antisolvent. **a**, In situ photoluminescence (PL) intensities of a precursor mixture for 3 monolayer (ML) nanorods, left to stir without injection of antisolvent. **b**, Extracted PL intensities from **a**. The emission of intermediate nanoclusters > 450 nm appears and remains visible throughout the entire duration of the experiment. Later, the emission < 450 nm of 2ML nanoplatelets becomes visible and finally dominates the spectrum. **c**, Ex situ absorption and PL of the purified reaction mixture reveals the characteristic optical features of monodisperse 2ML nanoplatelets (absorption at 429 nm, emission at 436 nm, full width at half maximum (FWHM) $= 12$ nm). Transmission electron microscopy (TEM) imaging in **d** confirms the nanoplatelet shape. The corresponding TEM image shows that nanoplatelets grow to a larger lateral size of (36 ± 5) nm than in the presence of antisolvent. **e**, In situ PL intensities of a precursor mixture for 2ML nanoplatelets, left to stir without injection of antisolvent. Compared to the 3ML nanorod reaction mixture the PL emission > 450 nm is lower. **f**, Extracted PL intensities from **e**. The emission < 450 nm, characteristic of 2ML nanoplatelets, grows in a linear fashion at a faster rate than the emission > 450 nm and finally dominates the spectrum. **g**, Ex situ absorption and PL of the purified product show monodisperse 2ML nanoplatelets (absorption at 428 nm, emission at 436 nm, FWHM $= 11$ nm). **h**, Corresponding TEM image. Again, nanoplatelets grow to a larger lateral size of (28 ± 3) nm than in the presence of antisolvent. In summary, for reactions without antisolvent and longer reaction times, 2ML nanoplatelets are observed, independent of precursor ratio. This indicates that the 2ML nanoplatelet shape is the thermodynamically stable polymorph in the Cs-deficient regime studied here.

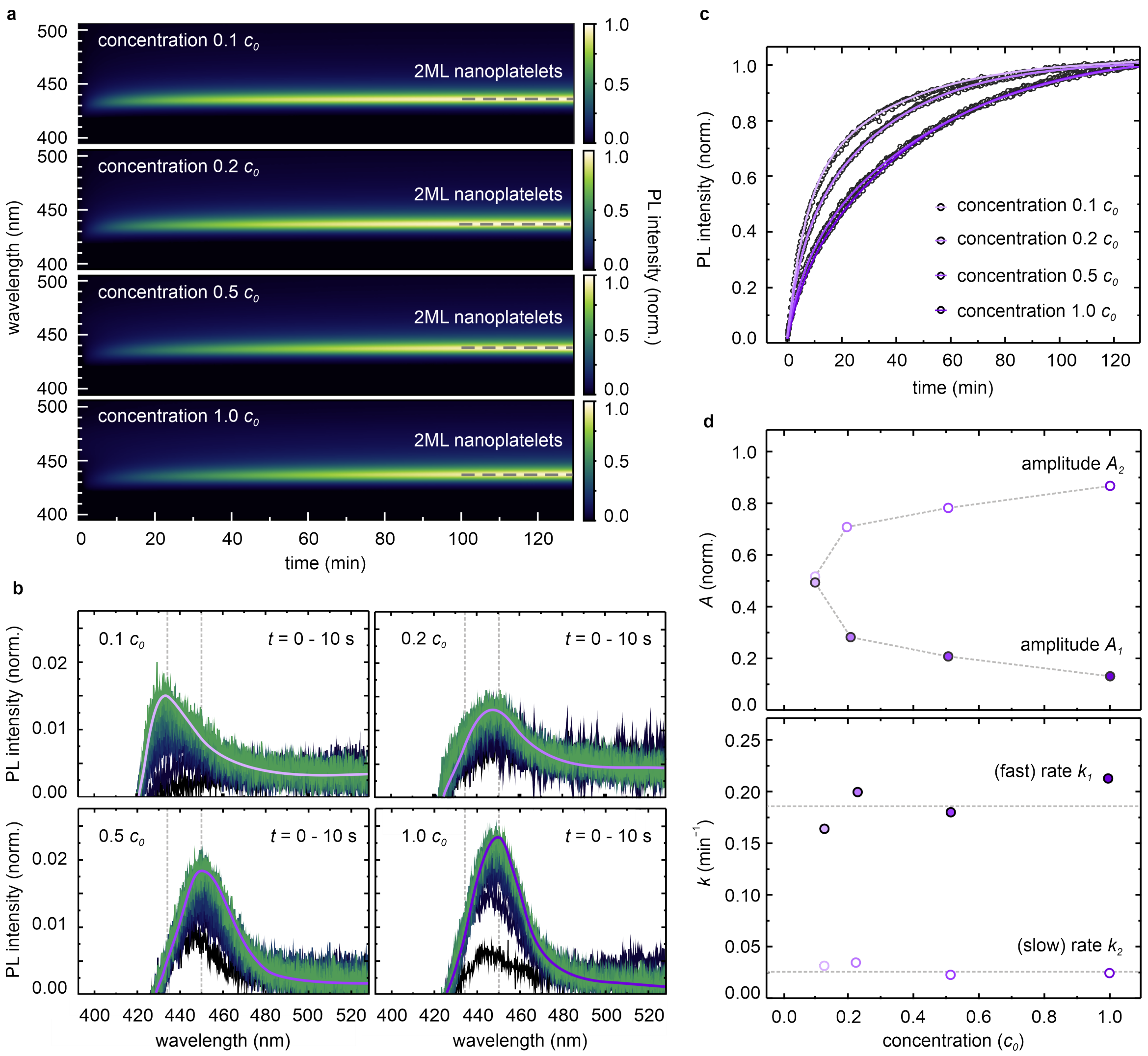

Supplementary Fig. 27 | In situ PL and growth kinetics of 2ML nanoplatelets synthesised without antisolvent at different precursor concentrations. **a**, In situ photoluminescence (PL) of 2 monolayer (ML) nanoplatelet syntheses at varying concentrations without antisolvent injection. **b**, Early stage PL spectra from $t = 0$ s to $t = 10$ s (purple lines) of syntheses shown in **a**. The PL emissions of 2ML nanoplatelets at 434 nm and of intermediate nanoclusters at 450 nm are marked for comparison (grey dashed lines). At standard concentration c_0, the emission of intermediate nanoclusters is observed after Cs-oleate injection. At decreasing precursor concentration, emission at 450 nm decreases and the PL maximum shifts to 432 nm, likely indicating that fewer intermediate nanoclusters form at lower precursor concentration. Ultimately, monodisperse 2ML nanoplatelets are obtained in all syntheses. **c**, Extracted PL intensities from **a**, fitted with a biphase exponential association function (purple lines) from $t = 0$ s to the point at which the PL intensity reaches a constant value and 2ML nanoplatelet growth is concluded. **d**, Rate constants and amplitudes extracted from the biphase exponential association fits of in situ PL in **c**. Overall, slightly faster growth of 2ML nanoplatelets is observed at lower precursor concentrations. Good fit results with a biphase exponential association function (Supplementary Table 10) hint at two different reaction pathways contributing to 2ML nanoplatelet formation. Faster growth with a rate k_1 proceeds from freely available precursors and growth with a rate k_2 from material supplied by dissolving intermediate nanoclusters. The extent to which each individual reaction pathway contributes to 2ML nanoplatelet growth is given by the amplitudes A_1 and A_2. In summary, the amount of freely available precursors is reduced due to the formation of intermediate nanoclusters at standard synthesis concentration. These act as reservoirs and gradually release precursor material again, therefore slowing down 2ML nanoplatelet growth compared to syntheses in which fewer intermediate nanoclusters form, i.e., at lower concentrations.

Extracted and normalized PL intensities of 2ML nanoplatelet syntheses at different precursor concentrations were fitted with a biphase exponential association function, with amplitudes A_1, A_2 and rates k_1, k_2.

$$I(t) = A_1 \cdot (1 - \mathrm{e}^{-k_1 t}) + A_2 \cdot (1 - \mathrm{e}^{-k_2 t}) \tag{S8}$$

Supplementary Table 10 | Biphase exponential association fit parameters for growth of 2ML nanoplatelets without antisolvent at different precursor concentrations.

concentration	A_1	k_1 (min^{-1})	A_2	k_2 (min^{-1})	R^2
0.1 c_0	0.4896 ± 0.0010	0.163 ± 0.002	0.5072 ± 0.0010	0.028 ± 0.007	0.9992
0.2 c_0	0.2835 ± 0.0009	0.200 ± 0.003	0.7131 ± 0.0008	0.031 ± 0.002	0.9992
0.5 c_0	0.2112 ± 0.0007	0.179 ± 0.005	0.7888 ± 0.0005	0.019 ± 0.009	0.9995
1.0 c_0	0.1303 ± 0.0007	0.211 ± 0.008	0.8688 ± 0.0005	0.021 ± 0.004	0.9994

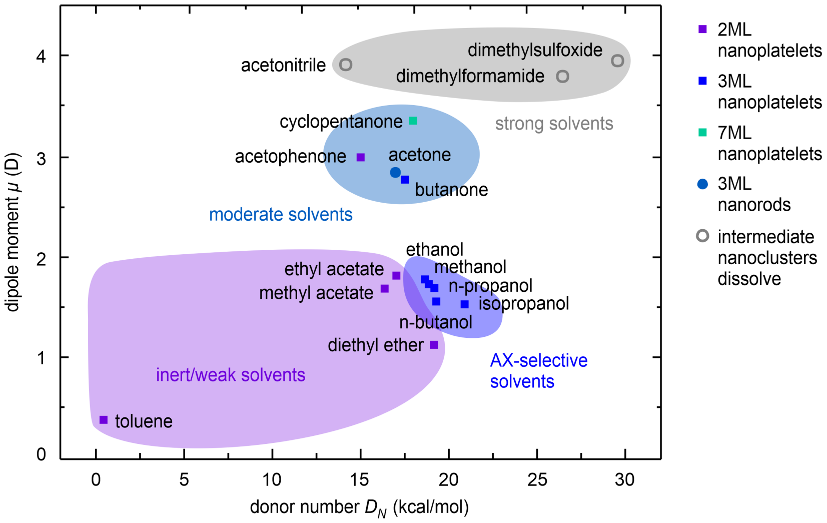

Supplementary Fig. 28 | Variation of antisolvent properties controls the dimensionality and thickness of anisotropic $CsPbBr_3$ nanocrystals. Shape anisotropy and monolayer (ML) thickness of perovskite nanocrystals classified by antisolvent properties (inert, weak, moderate, strong, and AX-selective) and given with respect to antisolvent dipole moment and donor number.

5 Characterisation of 3ML nanoplatelets

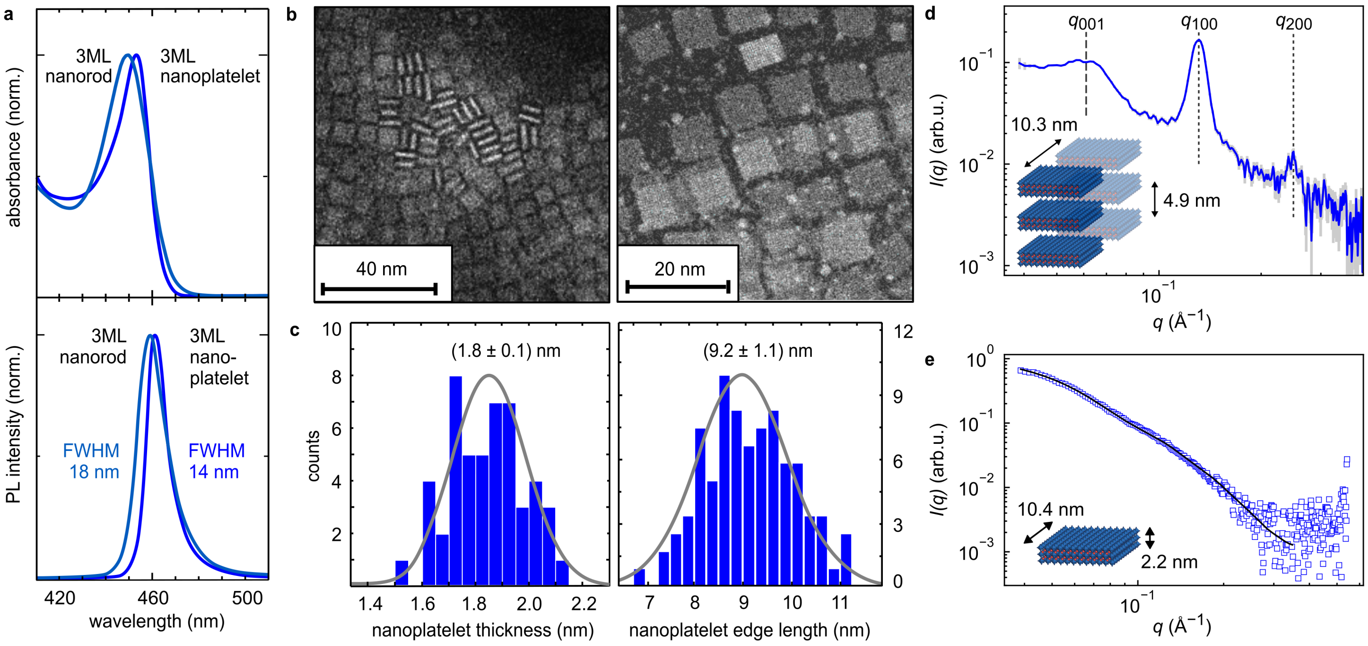

Supplementary Fig. 29 | Optical properties, morphology and assembly of 3ML $CsPbBr_3$ nanoplatelets. **a**, Absorbance and photoluminescence (PL) spectra of 3 monolayer (ML) nanoplatelets obtained through antisolvent engineering from syntheses with AX-selective solvents. Nanoplatelets exhibit a slightly redshifted emission and narrower linewidth (462 nm, full width at half maximum (FWHM) = 14 nm) compared to nanorods (460 nm, FWHM = 18 nm). **b**, Annular dark field scanning transmission electron microscopy (ADF-STEM) imaging of 3ML nanoplatelets shows both short stacks of edge-up oriented nanoplatelets as well as face-down oriented nanoplatelets. **c**, Distribution of platelet size obtained from transmission electron microscopy (TEM) images. 3ML nanoplatelets have a thickness of (1.8 ± 0.1) nm and square lateral dimensions of (9.2 ± 1.1) nm. **d**, Averaged small-angle X-ray scattering (SAXS) intensity of a 3ML nanoplatelet-forming reaction mixture after EtOH injection measured with a laboratory SAXS setup. 900 frames of 1 s duration were averaged. The inset depicts a sketch of the formed lamellar assembly in solution derived from the observed peak positions. **e**, SAXS intensity of purified 3ML nanoplatelets in n-hexane, measured with a laboratory setup. The black line is a fit with an ellipsoid model, which yields an an oblate shape $(2.2 \times 10.4 \times 10.4)$ nm^3. All fit parameters are given in Supplementary Table 1.

6 Antisolvent polarity and classification

Various antisolvents were tested in the ligand-assisted spontaneous crystallisation synthesis of anisotropic $CsPbBr_3$ nanocrystals and compared by relative polarity value $E_T(30)$[17,18], dipole moment μ[19], Hansen solubility parameter for hydrogen-bonding δ_H[20] and donor number D_N[21]. These parameters consider different types of interactions of antisolvent molecules with intermediate nanoclusters in the $PbBr_2$- and Cs-oleate precursor mixture, as well as with the eventual nanocrystal product of each synthesis. Based on these interactions, antisolvents were sorted into one of the following solvent categories[22] (Supplementary Table 11).

Inert solvents

This category includes nonpolar solvents like n-hexane or toluene, with very low values of dipole moment, Hansen hydrogen-bonding parameter and donor number, which are commonly used as solvents for ligand-passivated perovskite nanocrystals. These solvents do not demonstrate any chemical interaction with intermediate perovskite nanoclusters, nor with lead halide perovskites in general, and guarantee a decent stability of purified colloidal samples.

Weak solvents

Weak solvents like diethyl ether, methyl acetate and ethyl acetate possess a low polarity and small dipole moment, resulting in a slightly higher precursor solubility and weak ability to interact with intermediate perovskite nanoclusters. In comparison to inert solvents, they accelerate nanoplatelet formation and precipitation when used as antisolvents in a ligand-assisted spontaneous crystallisation synthesis approach.

Moderate solvents

This category is comprised of ketone solvents with moderate values of polarity, dipole moment and Hansen parameter for hydrogen-bonding, such as acetone and butanone.

Strong solvents

Strong solvents are moderately polar and specifically characterised by a large dipole moment and/or high donor number. They demonstrate a particular affinity for coordination of Pb^{2+} ions and dissolve preformed intermediate perovskite nanoclusters when injected as antisolvents.

AX-selective solvents

This category includes polar and protic solvents with very high Hansen hydrogen-bonding parameters but rather low dipole moments, namely various primary and secondary alcohols. These can effectively remove ligands from the surface of perovskite nanocrystals due to strong hydrogen bonding with the N-H groups of oleylamine, thereby enabling further growth of intermediate nanoclusters into 3ML nanoplatelets through attachment of precursor material.

Supplementary Table 11 | Polarity of antisolvents. Normalised polarity value, dipole moment, Hansen hydrogen-bonding parameter and donor number of various antisolvents. Antisolvents are sorted into categories with respect to their effect on the stability of intermediate nanoclusters and eventual perovskite nanocrystal products.

solvent	E_T(30)	μ (D)	δ_H (MPa)$^{1/2}$	D_N (kcal/mol)	classification
hexane	0.009	0.0	0.0	0.0	inert
toluene	0.099	0.375	2.0	0.1	inert
diethyl ether (DEE)	0.117	1.150	5.1	19.2	weak
ethyl acetate (EtOAc)	0.228	1.780	7.2	17.1	weak
methyl acetate (MeOAc)	0.253	1.720	7.6	16.3	weak
cyclopentanone	0.269	3.300	5.2	18.0	moderate
acetophenone	0.306	3.020	3.7	15.0	moderate
butanone	0.327	2.779	5.1	17.4	moderate
acetone	0.355	2.860	7.0	17.0	moderate
dimethylformamide (DMF)	0.386	3.820	11.3	26.6	strong
dimethylsulfoxide (DMSO)	0.444	3.960	10.2	29.8	strong
acetonitrile (ACN)	0.460	3.925	6.1	14.1	strong
isopropanol (i-PrOH)	0.546	1.580	16.4	21.1	AX-selective
n-butanol (n-BuOH)	0.586	1.660	15.8	19.5	AX-selective
n-propanol (n-PrOH)	0.617	1.650	17.4	19.8	AX-selective
ethanol (EtOH)	0.654	1.680	19.4	19.2	AX-selective
methanol (MeOH)	0.762	1.700	22.3	19.0	AX-selective

Supplementary Table 12 | Parameters for the ex situ synthesis of monodisperse $CsPbBr_3$ nanocrystals. Precursor volumes, Cs/$PbBr_2$ ratio, antisolvent type, volume, and respective nanocrystal product.

V_{PbBr2} (µL)	$V_{Cs-oleate}$ (µL)	Cs/$PbBr_2$	antisolvent	$V_{antisolvent}$ (µL)	product
3000	150	0.1	-	0	2ML nanoplatelets
1500	150	0.2	-	0	2ML nanoplatelets
3000	150	0.1	toluene	2000	2ML nanoplatelets
1500	150	0.2	toluene	2000	2ML nanoplatelets
3000	150	0.1	DEE	2000	2ML nanoplatelets
1500	150	0.2	DEE	2000	2ML nanoplatelets
3000	150	0.1	EtOAc	2000	2ML nanoplatelets
1500	150	0.2	EtOAc	2000	2ML nanoplatelets
3000	150	0.1	MeOAc	2000	2ML nanoplatelets
1500	150	0.2	MeOAc	2000	2ML nanoplatelets
3000	150	0.1	i-PrOH	500	2ML nanoplatelets
3000	150	0.1	n-BuOH	500	2ML nanoplatelets
3000	150	0.1	EtOH	250	2ML nanoplatelets
3000	150	0.1	EtOH	500	2ML nanoplatelets
3000	150	0.1	MeOH	250	2ML nanoplatelets
1500	150	0.2	acetophenone	2000	2ML nanoplatelets
1500	150	0.2	butanone	2000	3ML nanoplatelets
1200	300	0.5	butanone	2000	5ML nanorods
3000	150	0.1	acetone	500	2ML nanoplatelets
3000	150	0.1	acetone	1000	2ML nanoplatelets
3000	150	0.1	acetone	1500	2ML nanoplatelets
3000	150	0.1	acetone	2000	2ML nanoplatelets
1500	150	0.2	acetone	1000	3ML nanoplatelets
1500	150	0.2	acetone	1250	3ML nanoplatelets
1500	150	0.2	acetone	1500	3ML nanoplatelets
1500	150	0.2	acetone	1800	3ML nanorods
1500	150	0.2	acetone	1900	3ML nanorods
1500	150	0.2	acetone	2000	3ML nanorods
1500	150	0.2	acetone	2100	3ML nanorods
1500	150	0.2	acetone	2200	3ML nanorods
1500	150	0.2	cyclopentanone	2000	7ML nanoplatelets
1500	150	0.2	i-PrOH	2000	3ML nanoplatelets
1500	150	0.2	n-BuOH	2000	3ML nanoplatelets
1500	150	0.2	n-PrOH	2000	3ML nanoplatelets
3000	150	0.1	EtOH	2000	3ML nanoplatelets
1500	150	0.2	EtOH	250	3ML nanoplatelets
1500	150	0.2	EtOH	500	3ML nanoplatelets
1500	150	0.2	EtOH	750	3ML nanoplatelets
1500	150	0.2	EtOH	1000	3ML nanoplatelets
1500	150	0.2	EtOH	1250	3ML nanoplatelets
3000	150	0.1	MeOH	500	3ML nanoplatelets
3000	150	0.1	MeOH	750	3ML nanoplatelets
3000	150	0.1	MeOH	1000	3ML nanoplatelets
1500	150	0.2	MeOH	250	3ML nanoplatelets
1500	150	0.2	MeOH	500	3ML nanoplatelets
3000	150	0.1	DMF	2000	none
3000	150	0.1	DMF	2750	none
1500	150	0.2	DMF	1500	none
1500	150	0.2	DMF	2000	none
3000	150	0.1	DMSO	1750	none
3000	150	0.1	DMSO	2000	none
1500	150	0.2	DMSO	1000	none
1500	150	0.2	DMSO	2000	none
3000	150	0.1	ACN	1250	none
3000	150	0.1	ACN	2000	none
1500	150	0.2	ACN	750	none
1500	150	0.2	ACN	2000	none

7 Tuning of color, size, and aspect ratio

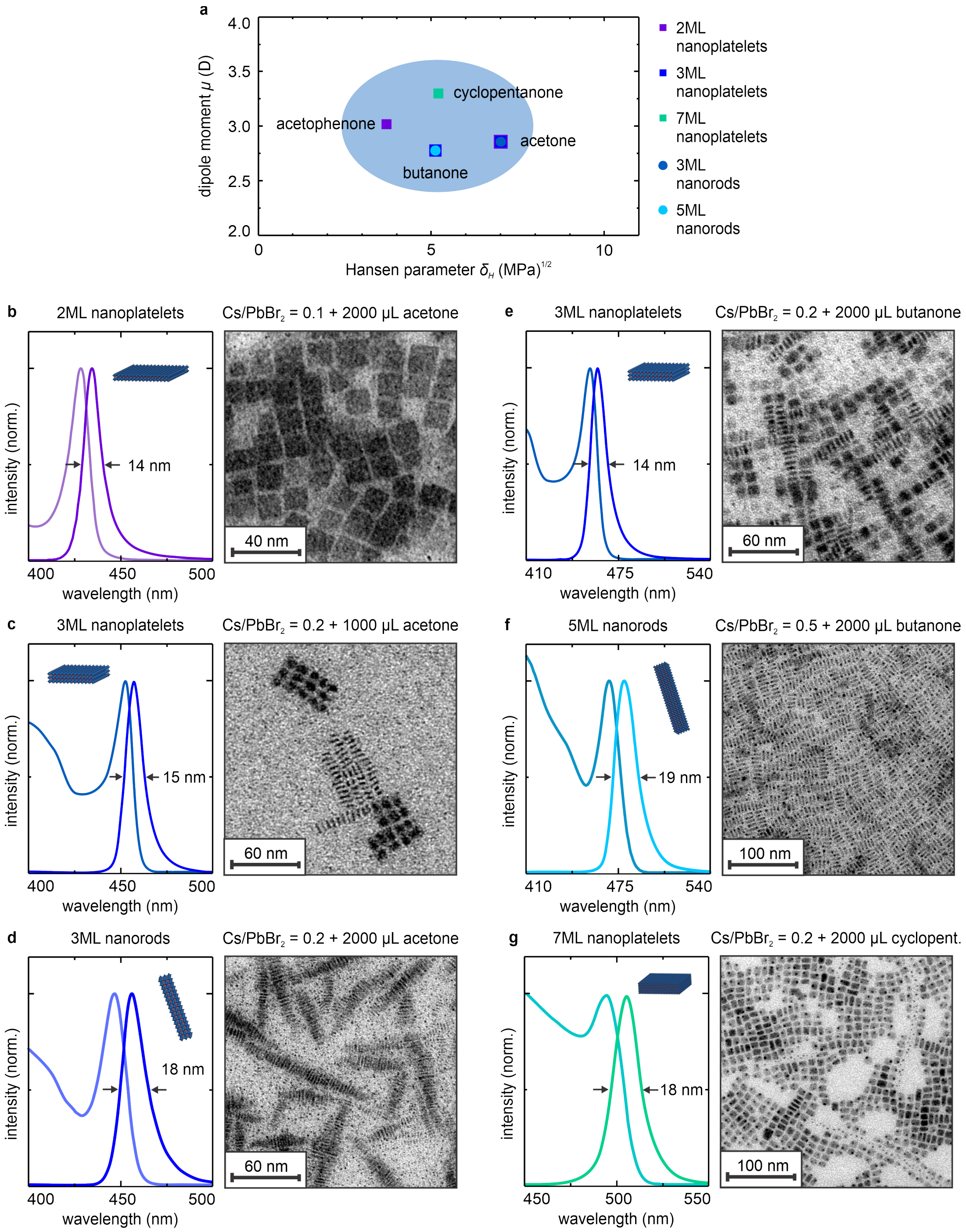

Supplementary Fig. 30 | Optical properties and morphology of $CsPbBr_3$ nanocrystals synthesised with various ketones as moderate antisolvents. **a**, Enlarged section of Fig. 5 b, showing ketone solvents with moderate dipole moment and Hansen hydrogen bonding parameter similar to acetone. Applying these antisolvents in the established synthesis procedure, a variety of perovskite nanocrystals with different sizes and shape anisotropy are obtained. **b**, Ex situ absorption spectra, photoluminescence (PL) spectra and transmission electron microscopy (TEM) images confirm the formation of monodisperse 2 monolayer (ML) nanoplatelets, **c**, 3ML nanoplatelets and **d**, 3ML nanorods in syntheses with Cs-oleate/$PbBr_2$ precursor = 0.1 - 0.2 and acetone as antisolvent. Both monodisperse perovskite nanoplatelets and nanorods are also accessible in syntheses with butanone as antisolvent, albeit at a ratio of Cs-oleate/$PbBr_2$ precursor = 0.2-0.5, as shown by the ex situ absorption spectra, PL spectra and TEM images of **e**, 3ML nanoplatelets and **f**, 5ML nanorods. A higher ratio of Cs-oleate/$PbBr_2$ = 0.5 could result in the formation of larger intermediate nanoclusters. Assuming that a similar assembly into a hexagonal mesophase occurs, a fusion of larger intermediate nanoclusters results in the formation of thicker, monodisperse 5ML nanorods with a narrow sky-blue PL centred at 480 nm (full width at half maximum (FWHM) = 19 nm). **g** Replacing acetone with cyclopentanone yields up to 7ML nanoplatelets with bright and narrow (FWHM = 18 nm) green emission at 503 nm.

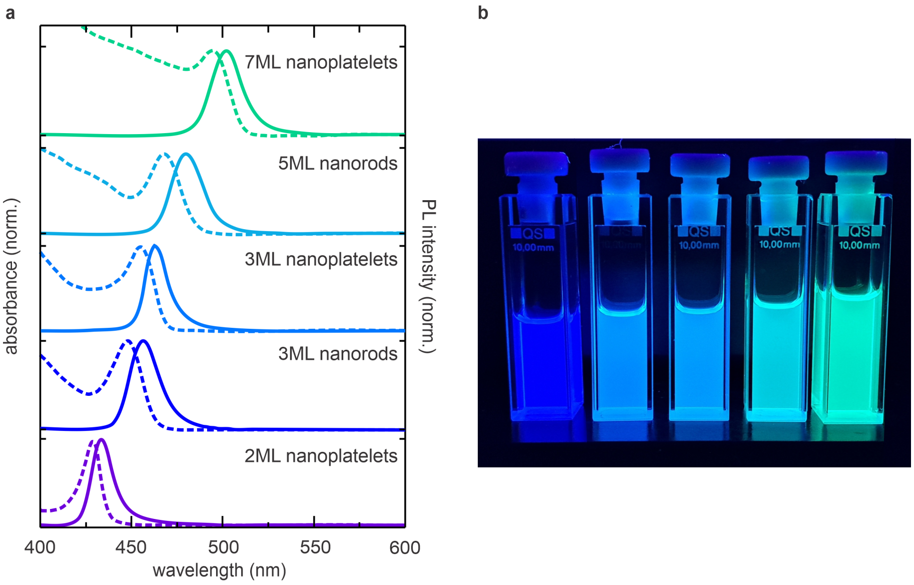

Supplementary Fig. 31 | Color tunability of perovskite nanocrystals obtained by antisolvent variation in room temperature synthesis. **a**, Anisotropic perovskite nanocrystals exhibit shape- and thickness-tunable absorption and emission covering the spectral range between 430 nm and 505 nm. **b**, Diluted colloidal samples of $CsPbBr_3$ nanocrystals in hexane under a UV lamp ($\lambda = 365$ nm).

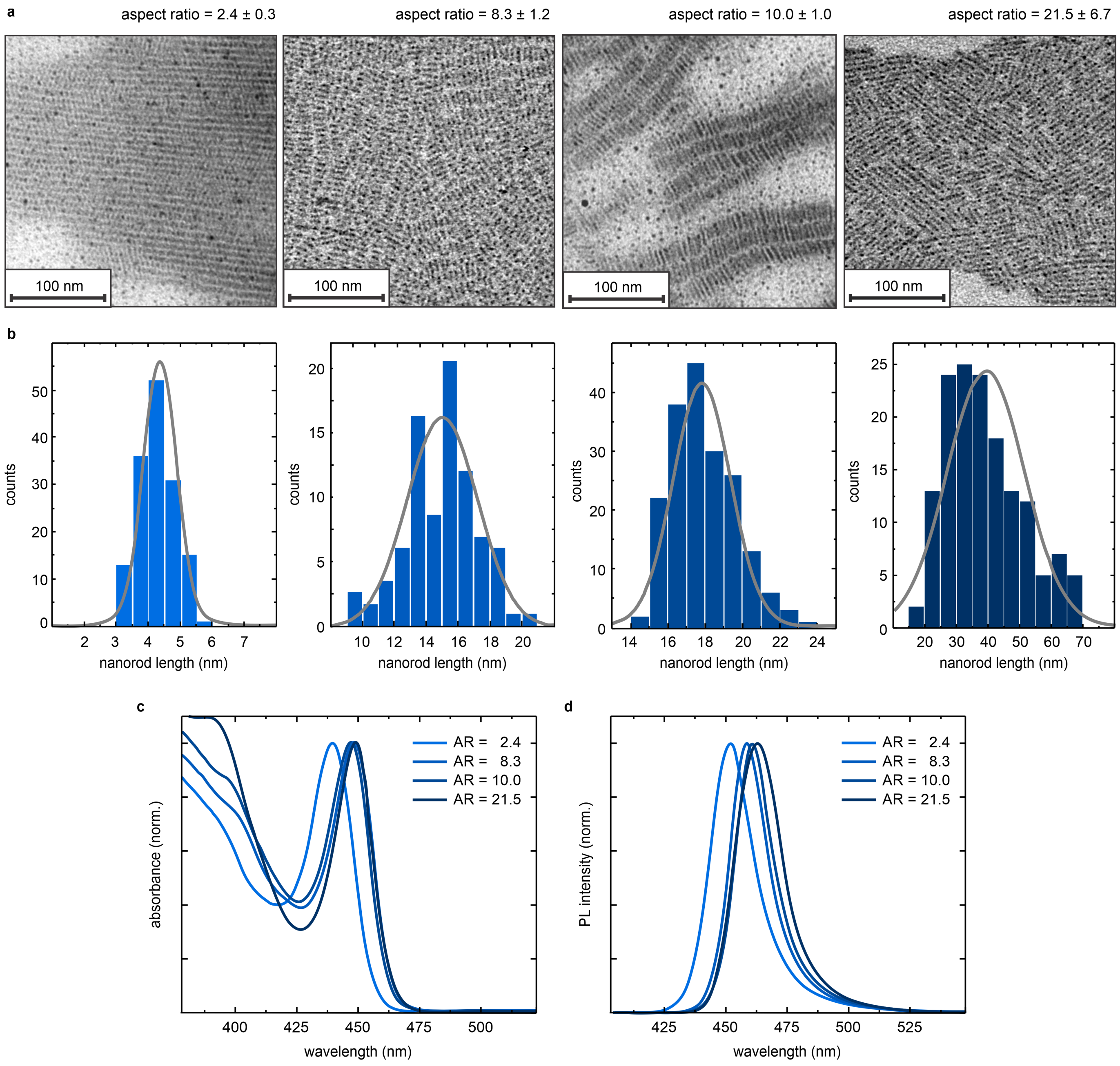

Supplementary Fig. 32 | Tunable aspect ratio of 3ML nanorods. a, Transmission electron microscopy (TEM) images of perovskite intermediate nanoclusters and 3 monolayer (ML) nanorods with different aspect ratio (AR), ranging from 2.4 ± 0.3 to 21.5 ± 6.7. **b**, Size distribution of $CsPbBr_3$ 3ML nanorod length. Intermediate perovskite nanoclusters were isolated in syntheses with weak antisolvents (MeAc, EtAc) and higher Cs-oleate/$PbBr_2$ precursor ratio. Nanorods with higer aspect ratio were obtained in syntheses with acetone and increasing concentration of the Cs-oleate precursor. **c**, Respective ex situ absorbance and **c**, Photoluminescence (PL) spectra of perovskite intermediate nanoclusters and 3ML nanorods shown in **a**. A redshift of excitonic absorption (437 nm to 449 nm) and PL (451 nm to 465 nm) is observed for increasing 3ML nanorod length and aspect ratio.

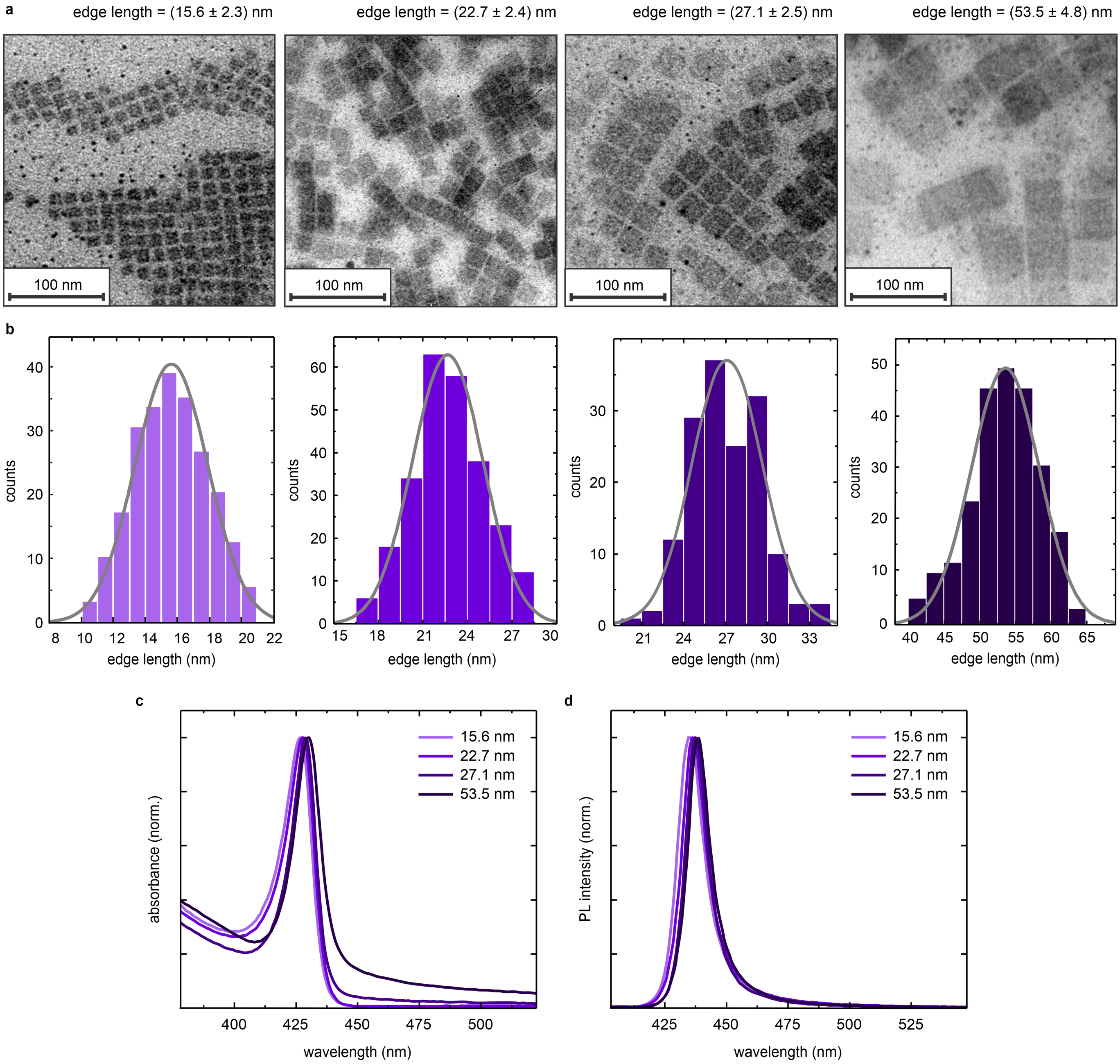

Supplementary Fig. 33 | Tunable lateral sizes of 2ML nanoplatelets. **a**, Transmission electron microscopy (TEM) images of 2 monolayer (ML) nanoplatelets with different lateral sizes, ranging from (15.6 ± 2.3) nm to (53.5 ± 4.8) nm. Nanoplatelets with larger lateral sizes were obtained in synthesis with lower polarity antisolvents (EtAc, MeAc) and slightly increased precursor ratio up to Cs-oleate/$PbBr_2$ = 0.2, resulting in overall longer reaction times. **b**, Size distribution of $CsPbBr_3$ 2ML nanoplatelets. **c**, Respective ex situ absorbance and **d**, Photoluminescence (PL) spectra of 2ML nanoplatelets shown in **a**. A slight redshift of excitonic absorption (426 nm to 428 nm) and PL (432 nm to 435 nm) is observed for increasing nanoplatelet edge length.

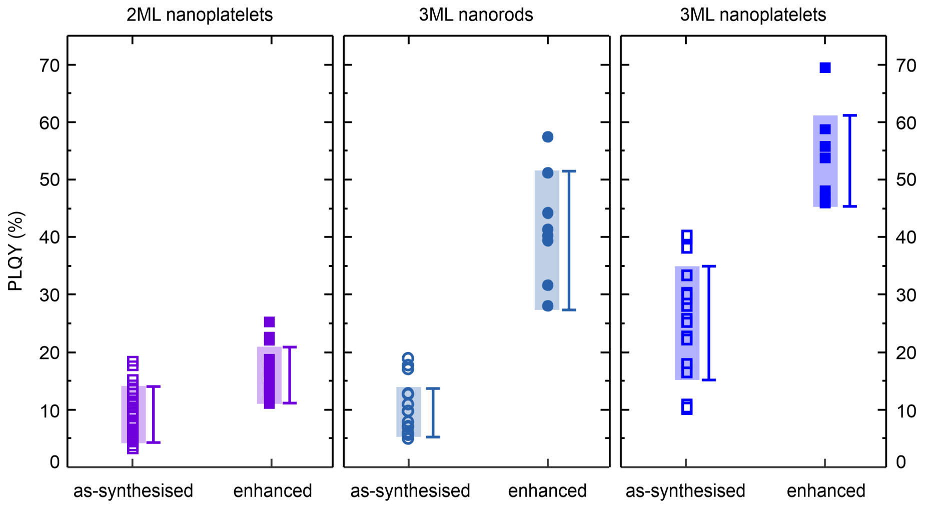

Supplementary Fig. 34 | Photoluminescence quantum yield of $CsPbBr_3$ nanoplatelets and nanorods. Absolute photoluminescence quantum yield (PLQY) of perovskite nanocrystals dispersed in n-hexane and measured with an integrating sphere. The average PLQY of as-synthesised 2 monolayer (ML) nanoplatelets is $(8.4 \pm 4.0)\,\%$, $(9.0 \pm 4.0)\,\%$ for 3ML nanorods, and $(25.3 \pm 10.1)\,\%$ for 3ML nanoplatelets. Post-synthetic treatment of as-synthesised samples with enhancement solution ($PbBr_2$, oleylamine, oleic acid dissolved in n-hexane) repairs surface defects and boosts PLQY to $(16.4 \pm 3.8)\,\%$, $(38.9 \pm 11.9)\,\%$, and $(53.1 \pm 7.7)\,\%$, respectively. Error bars denote the standard deviation.

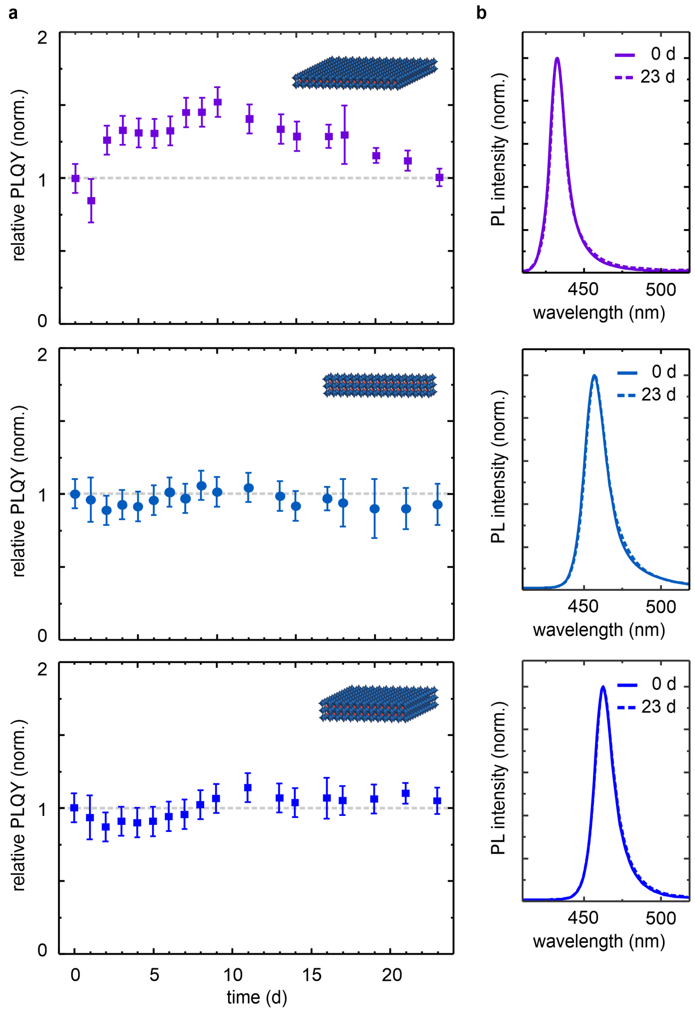

Supplementary Fig. 35 | Colloidal stability of perovskite nanoplatelets and nanorods. **a**, Relative photoluminescence quantum yield (PLQY) of 2 monolayer (ML) nanoplatelets, 3ML nanorods and 3ML nanoplatelets dispersed in n-hexane as a measure for stability. Values were calculated from the integrated photoluminescence (PL) intensity divided by the optical density (0.2) at the excitation wavelength ($\lambda = 400\,\text{nm}$) and normalised with respect to the value of the fresh sample (day 0). Error bars indicate the measurement precision and are derived from multiple consecutive measurements on the same day. Initial photoluminescence quantum yield is maintained throughout the observed timespan of 23 days, with 2ML nanoplatelets even showing a temporary improvement of up to 1.5. This could be attributed to self-healing of 2ML nanoplatelets or delayed passivation of nanoplatelet surface defects from excess precursor material in the sample. **b**, Respective PL spectra of fresh (day 0) and aged (day 23) colloidal samples shown in **a** with characteristic emission at 432 nm (2ML nanoplatelets), 460 nm (3ML nanorods) and 462 nm (3ML nanoplatelets). The absence of a significant spectral shift, broadening or appearance of additional peaks in the PL spectra of aged samples demonstrates excellent colloidal stability of perovskite nanoplatelets and nanorods.

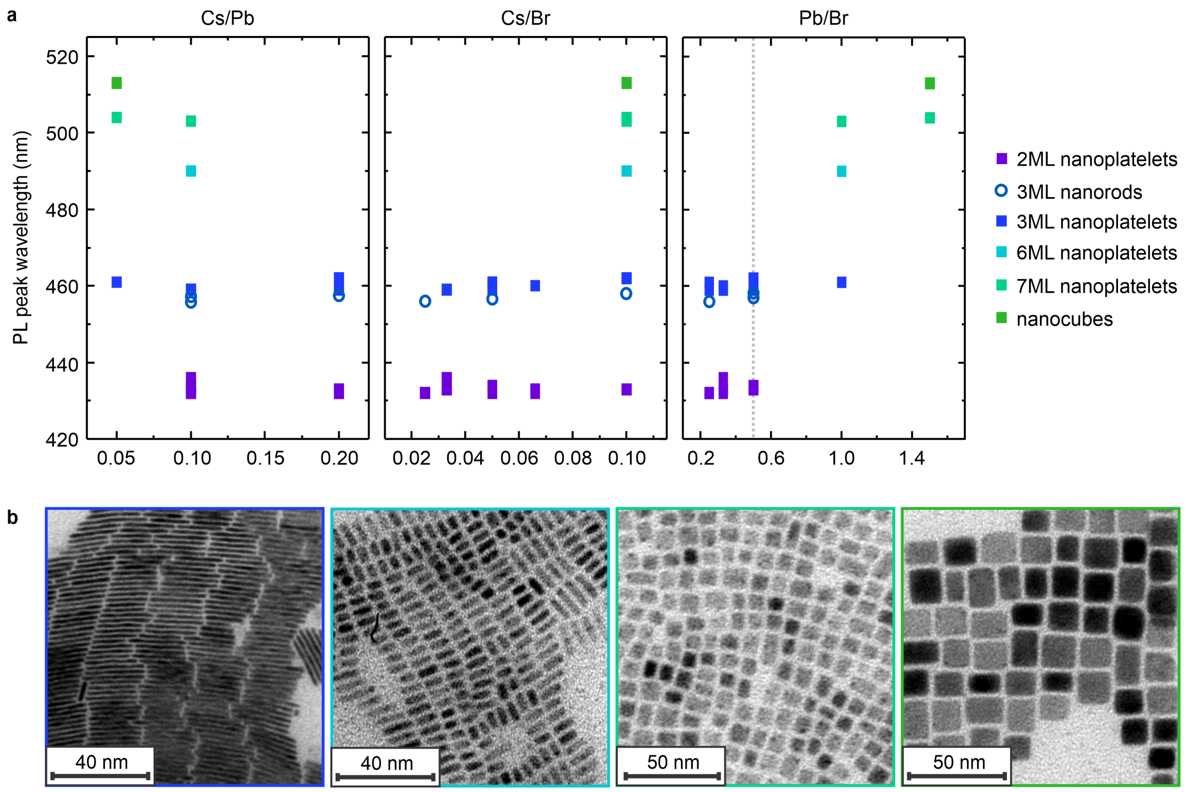

Supplementary Fig. 36 | Influence of Cs/Pb, Cs/Br and Pb/Br ratio on nanocrystal product. **a**, Photoluminescence (PL) emission wavelength of produced samples depending on Cs/Pb, Cs/Br and Pb/Br ratio in the synthesis. The grey dotted line marks the fixed stoichiometry of Pb/Br = 0.5 from the $PbBr_2$ precursor. Syntheses with varying Pb/Br ratio were realised by mixing separate precursors of Pb (Pb-oleate) or Br ($ZnBr_2$) with the standard $PbBr_2$ precursor solution before Cs-oleate is injected, followed by the antisolvent (acetone, MeOH) after 10-15 seconds. Within the Cs-deficient regime tested here, strongly confined 2 monolayer (ML) nanoplatelets, 3ML nanorods and 3ML nanoplatelets were obtained at many different Cs/Pb and Cs/Br ratios, but only in syntheses with lower Pb/Br ratio ≤ 1. Weakly-confined perovskite nanocrystals resulted from syntheses with lower Cs/Pb, but higher Cs/Br and Pb/Br ratios. Again, quasi-1D nanorods only formed in syntheses with acetone. Overall, the final nanocrystal product is determined by an intricate balance of multiple parameters, notably including antisolvent type and volume for shape control. **b**, Transmission electron microscopy (TEM) images of perovskite nanocrystals obtained in synthesis with varying Cs/Pb, Cs/Br and Pb/Br ratio, as shown in **a** and including 3ML nanoplatelets (blue), 5ML nanoplatelets (sky-blue) as well as weakly confined nanocrystals (mint, green) with green emission.

8 Chemicals

Supplementary Table 13 | Chemicals. List of chemicals, purity and supplier. All chemicals were used without purification.

**chemicals**	**chemical formula**	**purity**	**supplier**
acetone	C_3H_6O	$\geq$ 99.9%	VWR Chemicals
acetonitrile	CH_3CN	$\geq$ 99.5%	VWR Chemicals
acetophenone	C_8H_8O	for synthesis	Merck
butanone	C_4H_8O	$\geq$ 99%	VWR Chemicals
cyclopentanone	C_5H_8O	for synthesis	Merck
diethyl ether	$CH_5OC_2H_5$	$\geq$ 97.5%	Sigma Aldrich
dimethylformamide	$HCONC_2H_6$	for synthesis	Merck
dimethylsulfoxide	CH_3SOCH_3	$\geq$ 99.9%	Sigma Aldrich
n-butanol	C_4H_9OH	99%	Sigma Aldrich
cesium carbonate	Cs_2CO_3	97.5%	Sigma Aldrich
ethanol	C_2H_5OH	$\geq$ 99.5%	Sigma Aldrich
ethyl acetate	$C_4H_8O_2$	for synthesis	Merck
n-hexane	C_6H_{14}	$\geq$ 97%	VWR Chemicals
lanthanum hexaboride	LaB_6	powder, 10 µm, 99%	Sigma Aldrich
lead (II) acetate trihydrate	$C_4H_{12}O_7Pb$	$\geq$ 99.5%	VWR Chemicals
lead (II) bromide	$PbBr_2$	$\geq$ 98%	Sigma Aldrich
methanol	CH_3OH	$\geq$ 99.8%	Sigma Aldrich
methyl acetate	$C_3H_6O_2$	for synthesis	Merck
oleic acid	$C_{18}H_{34}O_2$	technical grade, 90%	Sigma Aldrich
oleylamine	$C_{18}H_{37}N$	technical grade, 70%	Sigma Aldrich
isopropanol	C_3H_7OH	for analysis	Merck
n-propanol	C_3H_7OH	$\geq$ 99%	VWR
silver behenate	$AgC_{22}H_{43}O_2$	$\geq$ 95%	ThermoFisher
toluene	$C_6H_5CH_3$	> 99.9%	VWR Chemicals
zinc bromide	$ZnBr_2$	> 99.9%	Sigma Aldrich

References

[1] Loubat, A. *et al.* Ultrathin gold nanowires: soft-templating versus liquid phase synthesis, a quantitative study. *J. Phys. Chem. C* **119**, 4422–4430 (2015).

[2] Macrae, C. F. *et al.* Mercury 4.0: From visualization to analysis, design and prediction. *J. Appl. Cryst.* **53**, 226–235 (2020).

[3] Gražulis, S. *et al.* Crystallography Open Database – an open-access collection of crystal structures. *J. Appl. Cryst.* **42**, 726–729 (2009).

[4] Juhás, P., Davis, T., Farrow, C. L. & Billinge, S. J. PDFgetX3: a rapid and highly automatable program for processing powder diffraction data into total scattering pair distribution functions. *J. Appl. Cryst.* **46**, 560–566 (2013).

[5] Terban, M. W. & Billinge, S. J. Structural analysis of molecular materials using the pair distribution function. *Chem. Rev.* **122**, 1208–1272 (2021).

[6] Stoumpos, C. C. *et al.* Crystal growth of the perovskite semiconductor $CsPbBr_3$: a new material for high-energy radiation detection. *Cryst. Growth Des.* **13**, 2722–2727 (2013).

[7] Lu, C. *et al.* Cesium oleate precursor preparation for lead halide perovskite nanocrystal synthesis: The influence of excess oleic acid on achieving solubility, conversion, and reproducibility. *Chem. Mater.* **31**, 62–67 (2018).

[8] Doucet, M. *et al.* Sasview version 5.0.5. Zenodo. (2022). Zenodo entry https://doi.org/10.5281/zenodo.6331344.

[9] Dahl, J. C. *et al.* Scientific machine learning of 2D perovskite nanosheet formation. *J. Am. Chem. Soc.* **145**, 23076–23087 (2023).

[10] Dahl, J. C. *et al.* Precursor chemistry of lead bromide perovskite nanocrystals. *ACS Nano* **18**, 22208–22219 (2024).

[11] Lumbreras, M., Protas, J., Jebbari, S., Dirksen, G. & Schoonman, J. Structure and ionic conductivity of mixed lead halides $PbCl_{2x}Br_{2(1-x)}$. II. *Solid State Ionics* **20**, 295–304 (1986).

[12] Shargaieva, O., Kuske, L., Rappich, J., Unger, E. & Nickel, N. H. Building blocks of hybrid perovskites: A photoluminescence study of lead-iodide solution species. *ChemPhysChem* **21**, 2327–2333 (2020).

[13] Juhás, P., Farrow, C., Yang, X., Knox, K. & Billinge, S. Complex modeling: a strategy and software program for combining multiple information sources to solve ill posed structure and nanostructure inverse problems. *Acta Cryst. A* **71**, 562–568 (2015).

[14] Dang, Z. *et al.* In situ transmission electron microscopy study of electron beam-induced transformations in colloidal cesium lead halide perovskite nanocrystals. *ACS Nano* **11**, 2124–2132 (2017).

[15] Glatter, O. & Kratky, O. *Small-Angle X-ray Scattering* (Academic Press, London, 1982).

[16] Newville, M. *et al.* lmfit version 1.2.2 Zenodo. (2023). Zenodo entry https://doi.org/10.5281/zenodo.8145703.

[17] Dimroth, K., Reichardt, C., Siepmann, T. & Bohlmann, F. Über Pyridinium-N-phenol-betaine und ihre Verwendung zur Charakterisierung der Polarität von Lösungsmitteln. *Liebigs Ann.* **661**, 1–37 (1963).

[18] Reichardt, C. Empirical parameters of the polarity of solvents. *Angew. Chem. Int. Ed.* **4**, 29–40 (1965).

[19] Lide, D. R. *CRC handbook of Chemistry and Physics, 84th Edition* (CRC Press, 2003).

[20] Hansen, C. M. *Hansen Solubility Parameters: A User's Handbook, Second Edition* (CRC Press, 2007).

[21] Laurence, C. & Gal, J.-F. *Lewis basicity and affinity scales* (Wiley-Blackwell, 2009).

[22] Tutantsev, A. S. *et al.* New pigeonholing approach for selection of solvents relevant to lead halide perovskite processing. *J. Phys. Chem. C* **124**, 11117–11123 (2020).
