# Supplementary figures and images for: Antisolvent controls the shape and size of anisotropic lead halide perovskite nanocrystals
Source: Nat Commun. 2024 Oct 17;15:8952. doi: 10.1038/s41467-024-53221-5 (PMC11486954; doi:10.1038/s41467-024-53221-5)

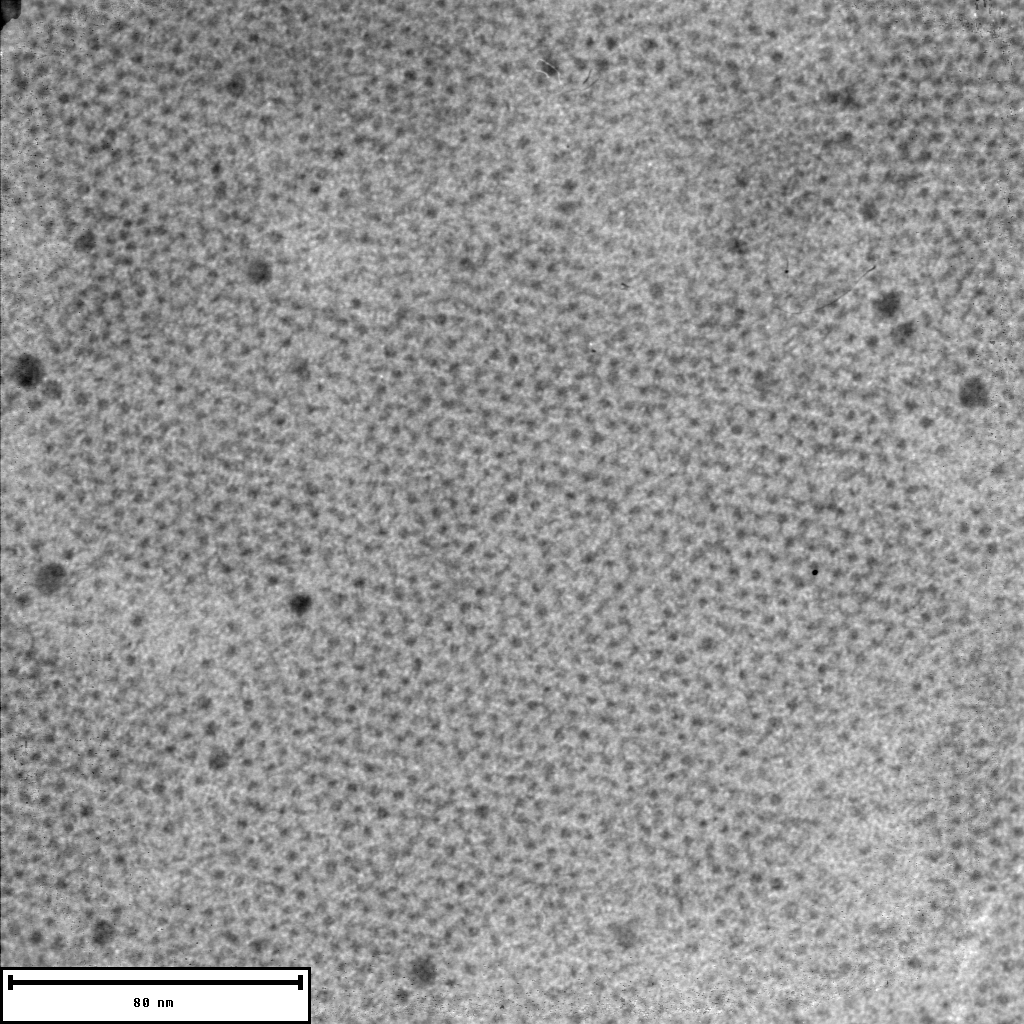

Supplement: Supplementary file 3 — Source Data [file 41467_2024_53221_MOESM3_ESM.zip › Fig03f_2ML_mix.tif]

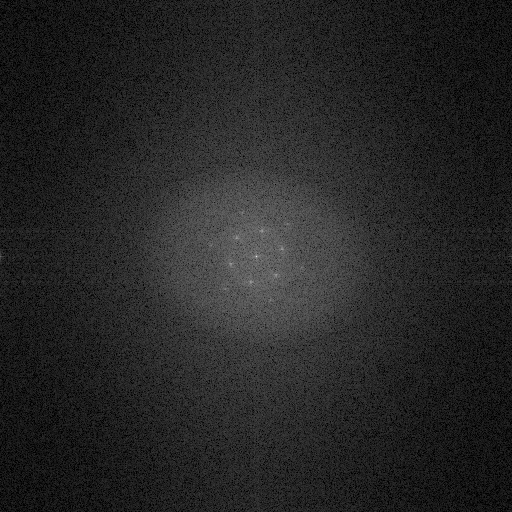

Supplement: Supplementary file 3 — Source Data [file 41467_2024_53221_MOESM3_ESM.zip › Fig03g_FFT_2ML_mix.tif]
